# Stabilization of High-Volume Circulating Fluidized Bed Fly Ash Composite Gravels via Gypsum-Enhanced Pressurized Flue Gas Heat Curing

**DOI:** 10.3390/ma18153436

**Published:** 2025-07-22

**Authors:** Nuo Xu, Rentuoya Sa, Yuqing He, Jun Guo, Yiheng Chen, Nana Wang, Yuchuan Feng, Suxia Ma

**Affiliations:** Shanxi Province Key Laboratory of Clean & High Efficient Combustion and Utilization of Circulating Fluidized Bed, Taiyuan University of Technology, 79 Yingze West Street, Taiyuan 030024, China; xunuo0322@link.tyut.edu.cn (N.X.); sarntoya@163.com (R.S.); 2024520613@link.tyut.edu.cn (Y.H.); 18334622464@163.com (J.G.); 18271758150@163.com (Y.C.); wangnana01@tyut.edu.cn (N.W.); fengyuchuan@tyut.edu.cn (Y.F.)

**Keywords:** composite gravel, circulating fluidized bed fly ash (CFBFA), pressurized flue gas heat curing, gypsum, life cycle assessment (LCA)

## Abstract

Circulating fluidized bed fly ash (CFBFA) stockpiles release alkaline dust, high-pH leachate, and secondary CO_2_/SO_2_—an environmental burden that exceeds 240 Mt yr^−1^ in China alone. Yet, barely 25% is recycled, because the high f-CaO/SO_3_ contents destabilize conventional cementitious products. Here, we presents a pressurized flue gas heat curing (FHC) route to bridge this scientific deficit, converting up to 85 wt% CFBFA into structural lightweight gravel. The gypsum dosage was optimized, and a 1:16 (gypsum/CFBFA) ratio delivered the best compromise between early ettringite nucleation and CO_2_-uptake capacity, yielding the highest overall quality. The optimal mix reaches 9.13 MPa 28-day crushing strength, 4.27% in situ CO_2_ uptake, 1.75 g cm^−3^ bulk density, and 3.59% water absorption. Multi-technique analyses (SEM, XRD, FTIR, TG-DTG, and MIP) show that FHC rapidly consumes expansive phases, suppresses undesirable granular-ettringite formation, and produces a dense calcite/needle-AFt skeleton. The FHC-treated CFBFA composite gravel demonstrates 30.43% higher crushing strength than JTG/TF20-2015 standards, accompanied by a water absorption rate 28.2% lower than recent studies. Its superior strength and durability highlight its potential as a low-carbon lightweight aggregate for structural engineering. A life-cycle inventory gives a cradle-to-gate energy demand of 1128 MJ t^−1^ and a process GWP of 226 kg CO_2_-eq t^−1^. Consequently, higher point-source emissions paired with immediate mineral sequestration translate into a low overall climate footprint and eliminate the need for CFBFA landfilling.

## 1. Introduction

The circulating fluidized bed (CFB) boiler, as a clean coal combustion technology, is widely used due to its many advantages, including wide coal combustion adaptability and low pollution. However, with the widespread use of CFB boilers, the proportion of CFB fly ash (CFBFA) in solid waste is increasing year by year. CFB boiler fly ash is a combustion product discharged from the flue of CFB boilers. The annual ash emission from CFB boilers in China is about 80 million tons [1]. CFBFA contains compounds such as Al_2_O_3_, SiO_2_, CaO, SO_3_, Fe_2_O_3_, and MgO, giving it both pozzolanic activity and self-hardening properties [2,3,4]. These self-hardening properties—i.e., the capacity to set and gain strength upon hydration—stem from the high contents of free CaO and anhydrite in CFBFA, which react with the aluminosilicate glass to generate Ca(OH)_2_, ettringite, and C–(A)–S–H gels that bind the ash particles together. These properties make CFBFA a valuable material for use in cement production [5,6], road base construction [7], concrete applications, artificial aggregates, and other building materials [8,9].

Currently, however, the comprehensive utilization rate of CFBFA remains notably low, with less than 25% of the total output being recycled [10,11,12]. The impact of CFBFA on the environment is multifaceted. Some of the heavy metal elements it contains, such as Zn, Pb, Cd, etc., if improperly disposed of, may be released into the soil and water bodies under the action of rainwater leaching, polluting the surrounding environment and affecting the ecosystem and human health [10,13,14]; the contained salts will increase the risk of soil salinization, damage the soil structure, and affect vegetation growth; and its fine particles are prone to generate dust during stacking and transportation, aggravating air pollution and affecting air quality [15,16,17,18,19].

The widespread use of natural gravel in road base construction has resulted in significant environmental impacts [20,21]. The over-extraction of these resources not only harms local ecosystems but also diminishes the availability of critical materials for future infrastructure projects. As a result, the need for a sustainable alternative, such as artificial gravel, has become increasingly urgent. This alternative must meet the standards set by the Technical Guide for Construction of Cement-Stabilized Macadam Base of Expressway (DB21/T 3873-2023) [22], with a compressive strength exceeding 4.0 MPa, as well as the Technical Guidelines for Construction of Highway Roadbases (JTG/TF20-2015) [23], which specify a strength range of 5.0–7.0 MPa.

The chemical composition of CFBFA is generally similar to that of ordinary fly ash. However, to enhance SO_2_ removal efficiency using sulfur-fixing agents like limestone or dolomite, the molar ratio of Ca/S in CFBFA is typically increased to 2.0–2.5. This leads to the production of a significant amount of f-CaO and the desulfurization product CaSO_4_ [2]. As a result, the content of CaO and SO_3_ in CFBFA is higher than in ordinary fly ash [1]. Due to the presence of CaO and SO_3_ in CFBFA, where SO_3_ primarily exists in the form of II-CaSO_4_, CFBFA exhibits expansibility [9]. The hydration of f-CaO results in the formation of Ca(OH)_2_, which induces volume expansion. He et al. [24] tested the linear expansion rate of CFBFA and found that f-CaO had a greater impact on the expansion rate than SO_3_. The linear expansion rate increased as the f-CaO content increased. The expansion-induced internal stress can weaken the material’s mechanical properties, leading to a reduction in overall structural performance. This is why the utilization of CFBFA is challenging. To improve material stability, common methods include grinding CFBFA [25] or controlling its dosage in cementitious materials [6]. Although previous research has contributed to the safe disposal of CFBFA, its utilization rate still remains under 20% [26]. Moreover, grinding into ultrafine powder results in significant energy consumption, increasing overall production costs [27]. Therefore, it is crucial to develop a solution that can both enhance material stability and enable low-cost, large-scale utilization of CFBFA.

Researchers have explored how different curing environments impact the expansion behavior of CFBFA-containing cementitious materials. For example, under standard curing conditions, the expansion rate of hardened cement paste with CFBFA was found to be related to the ash content [28]. Also, the influence of curing conditions on the performance of grout with CFBFA replacement, such as water demand, compressive strength, and expansion rate, has been investigated [29]. The role of standard curing time and sulfur content in CFBFA solidified bodies on expansion-related properties has also been a subject of research [30]. Additionally, the mechanism of CFBFA affecting Portland cement hydration under different curing conditions, including sealed and air-cured environments, has been revealed [31].

Wang et al. [32] found that gypsum dihydrate promotes the rapid hydration of steel slag to produce ettringite during the carbonation of steel slag, which in turn reacts with CO_2_ to produce the carbonation product carbonate. Gypsum plays a catalytic role in this carbonization reaction system. Ren et al. [7] observed that gypsum acted as a catalyst to promote the formation of hydration products and carbonation products in carbonated CFBFA-based cementitious materials. Gypsum is also considered an expansion source in cementitious materials, which limits the use of solid waste desulfurization gypsum [33,34]. The hydration of gypsum in cementitious systems leads to the formation of ettringite, a process that causes volume expansion and can negatively affect the mechanical properties and long-term durability of the material.

Based on recent findings, further research is required to elucidate the stability effects of gypsum in CFBFA cementitious systems—particularly under novel curing regimes such as pressurized flue gas heat curing (FHC). While previous studies have shown that carbonation curing can enhance the mechanical strength of cementitious materials, its influence on the stability of CFBFA-based systems remains insufficiently explored. Moreover, most existing research utilizes CFBFA at substitution rates below 20%, which greatly limits its resource utilization potential. Investigations into the synergistic effects of gypsum addition and pressurized flue gas heat curing in CFBFA-rich cementitious matrices remain extremely limited. Unraveling these mechanisms is crucial for optimizing the curing process and ensuring the mechanical stability of high-volume CFBFA composites when utilizing cost-effective CO_2_ sources such as flue gas.

In this study, a novel pressurized flue gas heat curing (FHC) approach was adopted to prepare CFBFA-based composite gravels containing a high proportion of solid waste (56.7–85% CFBFA). Gypsum was used not only as a hydration accelerator but also as a promoter of carbonation reactions. Curing was performed at 80 °C and 1 MPa in a simulated flue gas environment (15% CO_2_ and 85% N_2_), replicating the actual operational conditions in coal-fired power plants. The effects of gypsum content on the mechanical properties, stability, and microstructure of the composite gravels were systematically investigated. Key physical properties—including crushing strength, apparent density, and water absorption—were evaluated. Mercury intrusion porosimetry (MIP) was employed to analyze pore structure, while scanning electron microscopy (SEM), thermogravimetric analysis (TGA), X-ray diffraction (XRD), and Fourier-transform infrared spectroscopy (FTIR) provided insights into microstructural evolution, thermal stability, phase transformations, and chemical bonding, respectively. Kinetic analyses further elucidated the underlying hydration–carbonation mechanisms. By optimizing the gypsum-to-CFBFA ratio, significant improvements in material stability were demonstrated. This comprehensive, multi-technique investigation provides a holistic view of how pressurized flue gas heat curing—when combined with gypsum activation—can unlock the stability and performance of high-CFBFA composites. This study also established an integrated demonstration system (Jinneng Datuhe Thermal Power Plant) for the joint utilization of CFBFA, desulfurization gypsum, and flue gas CO_2_, and conducted a life cycle assessment (LCA) to rigorously evaluate the environmental benefits of this novel curing strategy. The fundamental mechanism by which gypsum dosage controls hydration–carbonation coupling and expansion in high-volume CFBFA matrices under pressurized flue gas heat curing remains unclear. No low-carbon artificial gravel currently valorizes large quantities of CFBFA and flue gas CO_2_ while meeting highway base strength requirements. Therefore, this study addresses both gaps by clarifying the gypsum-mediated mechanism and demonstrating a highway-grade, FHC-activated CFBFA composite gravel as a scalable alternative to natural aggregates.

## 2. Experiment

**Figure 1:**
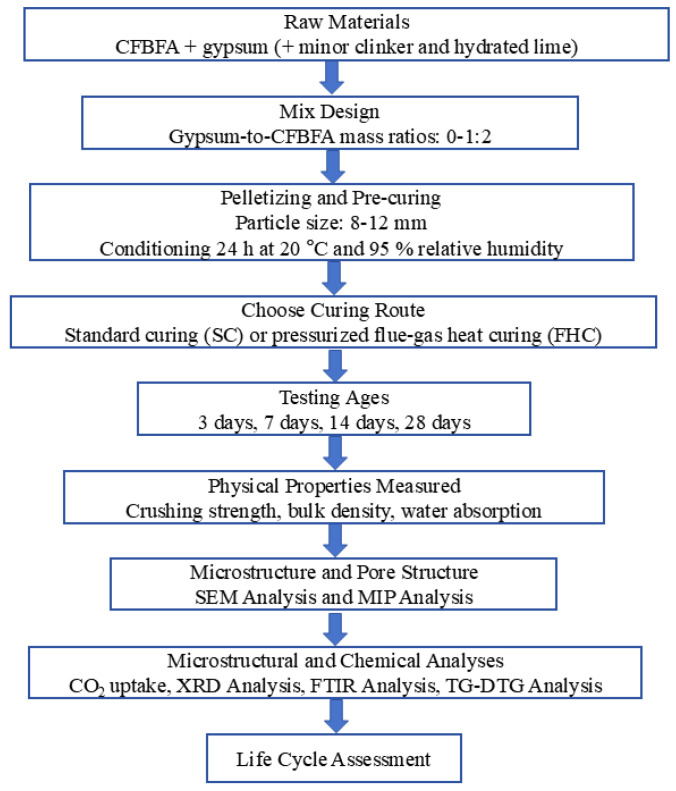
Schematic of experimental workflow and data parameters.

In the Experiment section, we present a flowchart that outlines the experimental research program. This flowchart provides an overview of the key factors, sample parameters, and data parameters involved in the study. It aims to guide the reader through the experimental setup and procedures, illustrating the sequence of actions from the preparation of materials to the testing and analysis stages (Figure 1).

### 2.1. Raw Materials

This experiment utilizes Portland cement (P.O. 42.5), CFBFA, gypsum, and hydrated lime as raw materials. CFBFA was supplied by Shanxi Jinneng Datuhe Thermal Power Co., Ltd. (Jinzhong, China), and Portland cement (PO 42.5) was provided by Jiuqi Building Materials Co., Ltd. (Linyi, China). The compressive strength of Portland cement is 17 MPa at 3 days and 42.5 MPa at 28 days, while the flexural strength is 3.5 MPa at 3 days and 6.5 MPa at 28 days. Gypsum (CAS: 10101-41-4, 99%) was purchased from Shanghai Aladdin Biochemical Technology Co., Ltd., Shanghai, China, and hydrated lime (CAS: 1305-62-0, 95%) was sourced from Shanghai Macklin Biochemical Technology Co., Ltd., Shanghai, China. Table 1 provides the chemical compositions of the admixtures used.

**Figure 2:**
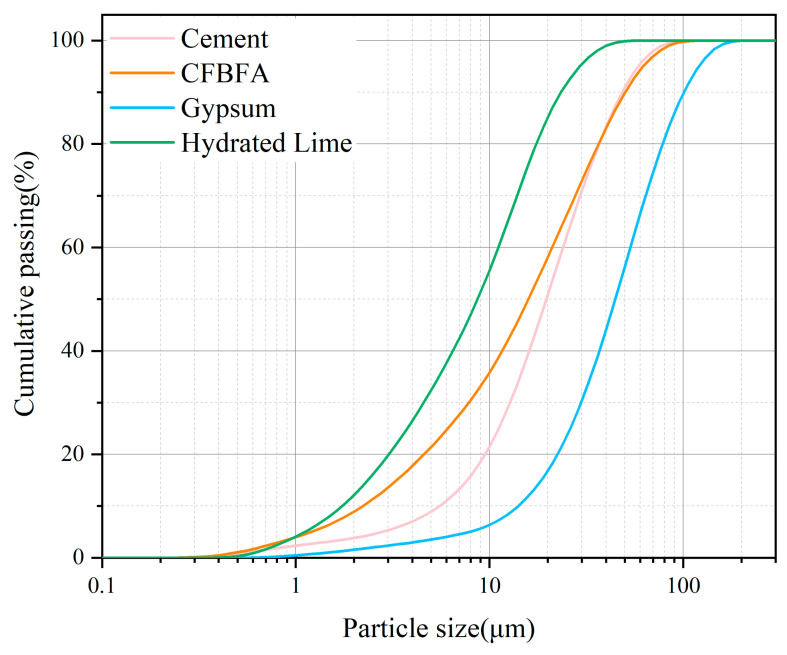
Particle size distribution of the raw materials measured by laser granulometry.

**Figure 3:**
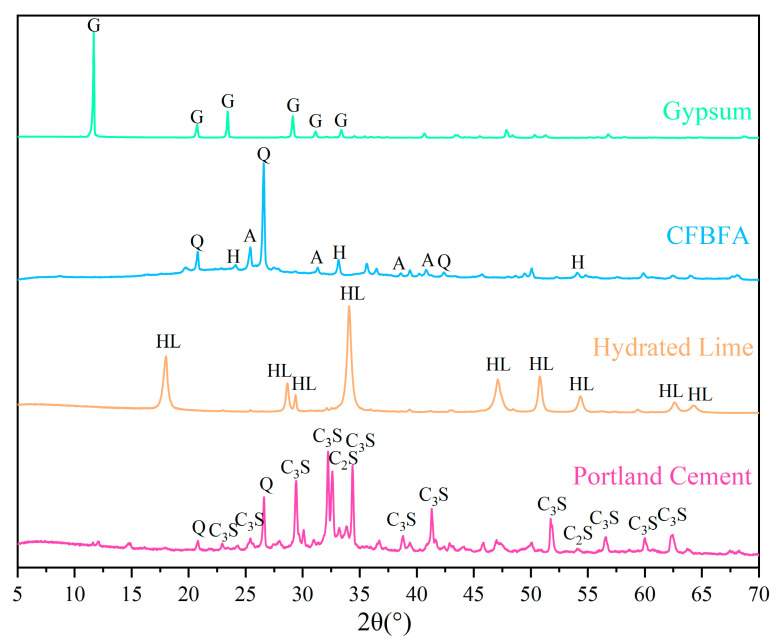
XRD pattern for raw materials (Q: Quartz; H: Hematite; and G: Gypsum; A: Anhydrite; HL: Hydrated Lime; C_3_S: Tricalcium Silicate; C_2_S: Dicalcium Silicate).

The chemical compositions of CFBFA, Portland cement, gypsum, and hydrated lime were determined using X-ray fluorescence (XRF), with the results presented in Table 1. The particle size distributions of these materials were measured using a laser particle size analyzer (BT-9300HT, Bettersize, Dandong, China), as shown in Table 1 and Figure 2. The specific surface areas of CFBFA, Portland cement, gypsum, and hydrated lime were found to be 333.6, 213.0, 84.95, and 974.5 m^2^/kg, respectively. The corresponding median diameters of these materials were 16.98 μm for CFBFA, 21.89 μm for Portland cement, 49.66 μm for gypsum, and 9.663 μm for hydrated lime. To confirm the intrinsic hydraulic activity of the raw CFBFA, we conducted preliminary tests by mixing it with deionized water only. The resulting paste exhibited low strength after 28 days of curing under standard conditions, indicating that external activators or curing strategies are essential for enhancing its performance. X-ray diffraction (XRD) analysis was performed using a Rigaku Ultima IV diffractometer (40 kV, 40 mA) (Tokyo, Japan) with a scanning range of 5–70° (2θ), a sampling width of 0.02°, and a scanning speed of 3°/min. The XRD patterns of the raw materials are shown in Figure 3. The main crystalline phases detected in CFBFA included quartz (SiO_2_), anhydrite (CaSO_4_), hematite (Fe_2_O_3_), and calcite (CaCO_3_). Cement primarily contained quartz (SiO_2_), hatrurite (C_3_S), and larnite (C_2_S), while gypsum was mainly composed of CaSO_4_·2H_2_O. Hydrated lime consisted predominantly of Ca(OH)_2_.

### 2.2. Proportion of Preparation and Curing

**Figure 4:**
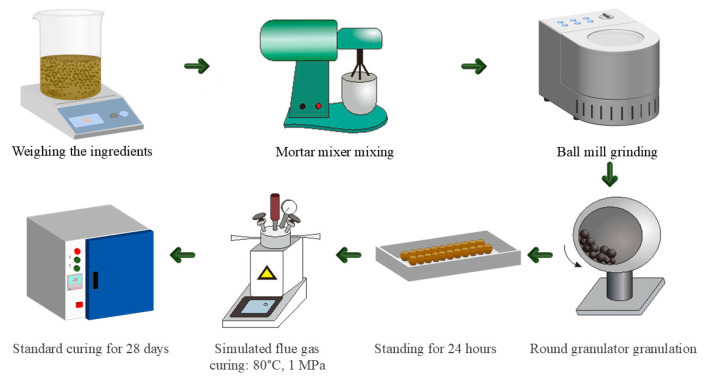
CFBFA composite gravels preparation process diagram.

In Table 2, the sample naming convention is in the form of GXFY. GXFY refers to the ratio of gypsum to CFBFA, where X represents the amount of gypsum and Y represents the amount of CFBFA. For instance, G1F2 means the ratio of gypsum to CFBFA is 1:2. G0F1 indicates that no gypsum is used. The feedstock ratios for CFBFA-based composite gravel were designed under two different curing conditions. Specifically, GXFYSC means the sample was cured under SC (28 d), which refers to the standard curing condition for 28 days, commonly applied to composite gravels. GXFYFHC means the sample underwent a two-stage curing process: first FHC (1 h), followed by SC (28 d). FHC refers to the pressurized flue gas heat curing process (for 1 h), designed to accelerate early hydration and enhance the early strength of the material. After this, the sample was further cured under SC (28 d) for 28 days. The samples were cured under the same two conditions, with varying amounts of gypsum and CFBFA in each sample. Figure 4 illustrates the experimental process for preparing CFBFA composite gravels, including weighing, mixing, ball milling, granulation, and curing under simulated flue gas. The raw materials—CFBFA, gypsum, Portland cement (P.O. 42.5), and hydrated lime—were proportioned according to the ratios in Table 2. After 5 min of mixing, the materials were ball-milled for 10 min and granulated with a water-to-solid ratio of 0.35 using tap water. The raw material was then formed into spherical pellets through the granulation process. After granulation, the pellets were allowed to rest for 24 h to stabilize the gravels before the curing process.

The composite gravels were prepared using a two-stage curing process. In the first stage, the samples underwent pressurized flue gas heat curing in an autoclave set to 80 °C and 1 MPa, with a gas mixture of 15% CO_2_ and 85% N_2_, simulating flue gas for one hour. The curing temperature (80 °C) and total pressure (1 MPa; pCO_2_ ≈ 0.15 MPa) were chosen on the basis of (i) real flue gas conditions at CFB power plants and (ii) a short series of preliminary screening tests.

Following the pressurized flue gas heat curing (FHC), the composite gravels were subjected to standard curing (SC). This stage was conducted at a controlled temperature of 20 ± 1 °C and a relative humidity above 95%. The gravels were allowed to cure under these conditions for 28 days.

Additionally, some of the composite gravels were cured directly using standard curing without undergoing the pressurized flue gas heat curing stage. These samples were exposed to the same standard curing conditions for 28 days to compare the effects of pressurized flue gas heat curing and standard curing on the properties of the composite gravels.

### 2.3. Methods

#### 2.3.1. Crushing Strength Test

The crushing strength of the aggregates was determined using a California Bearing Ratio tester. The crushing strength result of an individual aggregate was calculated according to Equation (1), and the average value of 12 composite gravels was reported. The equation is given as follows:

(1) $$\sigma =\frac{2.8P}{\pi d^{2}}$$

where σ is the crushing strength of the aggregate (MPa), P is the maximum load at failure (N), and d is the span length in the three-point bending test (mm).

#### 2.3.2. CO_2_ Absorption Performance

In this study, the absorption behavior of composite gravels was investigated using the H-Sorb X600 high-temperature and high-pressure gas absorption instrument (Guoyi Quantum Technology (Hefei) Co., Ltd., Hefei, Anhui Province, China) under isothermal and isobaric conditions. The instrument precisely measures the amount of carbon dioxide absorbed by the sample under controlled high-temperature and high-pressure conditions. During the experiment, composite gravel samples were placed in the instrument and exposed to carbon dioxide from simulated flue gas. The absorption process, involving the interaction between the hydration products of the composite gravels and carbon dioxide, was monitored by tracking the change in the amount of carbon dioxide absorbed over time.

#### 2.3.3. Apparent Density Test

**Figure 5:**
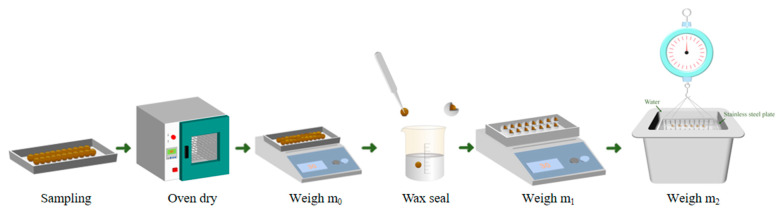
Flowchart of apparent density measurement procedure.

The method for testing apparent density is shown in Figure 5. First, samples of composite gravels are taken and then dried in an oven until they reach a constant weight. After drying, the mass of the sample is weighed and recorded as $m_{0}$. Next, the sample is sealed with wax to prevent water from entering its pores, and the mass of the wax-sealed sample is weighed and recorded as $m_{1}$. Finally, the wax-sealed sample is immersed in water, and its mass in water is measured and recorded as $m_{2}$.

(2) $$V_{0}={(m}_{1}-m_{2})-\frac{m_{1}-m_{2}}{\rho_{0}}$$

(3) $$\rho_{0}=\frac{m_{0}}{V_{0}}$$

where *m*_0_ is the mass of the sample after drying, *m*_1_ is the mass of the sample after wax sealing, and *m*_2_ is the mass of the wax-sealed sample weighed using the basket method.

The volume $V_{0}$ is calculated using Equation (2), and the apparent density $\rho_{0}$ is determined using Equation (3).

#### 2.3.4. Water Absorption Test

The 24 h water absorption was tested following the standard ASTM C127 [35], which involves immersing the aggregate in water for 24 h and measuring the amount of water it absorbs.

#### 2.3.5. Microstructural Analysis

XRD, TGA, SEM-EDS, FTIR, and MIP analyses were conducted to evaluate the impact of various factors on the chemical and physical properties of the specimens. X-ray diffraction (XRD) was performed using a Rigaku Ultima IV with a CuKα source, scanning from 5° to 70° (2θ) at a 0.02° sampling width and a scanning speed of 5°/min, with the generator set at 40 kV/40 mA. Prior to XRD testing, the sample was pulverized into a fine powder, ensuring uniform particle size, and then dried to remove moisture. Thermogravimetric analysis (TGA) was carried out on a NETZSCH (Selb, Germany). An appropriate amount of powder sample (30 mg) was weighed, and volatile components were removed at room temperature before testing. The microstructure was analyzed via scanning electron microscopy with energy dispersive spectrometry (SEM-EDS) using a JEOL JSM-IT200 (JEOL Ltd., Tokyo, Japan). For SEM, block-shaped composite particles were selected, and a conductive metal coating was applied to enhance imaging quality. Fourier transform infrared (FTIR) spectroscopy was performed on a Bruker TENSOR II (Bruker Optik GmbH, Ettlingen, Germany), covering a wavenumber range from 400 cm^−1^ to 4000 cm^−1^ to identify functional group characteristics. Mercury intrusion porosimetry (MIP) tests using a PoreMaster 33 (Quantachrome Instruments, Boynton Beach, FL, USA) introduced mercury at 0–30 psi, increased pressure to 31,000 psi, and then released it to measure porosity. Prior to MIP testing, samples were dried and vacuumed to remove internal gases and moisture, ensuring accurate results.

## 3. Results and Discussion

### 3.1. Physical Properties

#### 3.1.1. Crushing Strength

**Figure 6:**
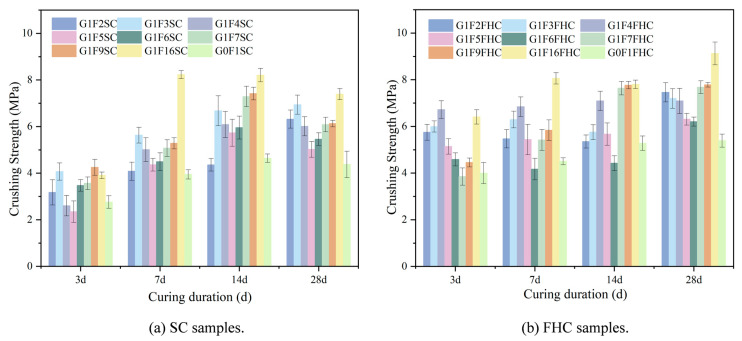
The crushing strength of SC and FHC samples at different ages.

In Figure 6a, the crushing strength data for each curing period (3 d, 7 d, 14 d, and 28 d) exhibit an M-shaped trend with two distinct peaks. At 3 days, G1F3SC and G1F16SC show the highest strength, with G1F3SC reaching the peak, indicating rapid early-stage strength development, while G1F6SC demonstrates the lowest strength. By 7 days, G1F16SC experiences a significant strength increase, forming the second peak of the M-shape and achieving its highest strength at this stage, while G0F1SC shows the lowest strength. G1F3SC forms the first peak, with both G1F3SC and G1F16SC exhibiting the two highest crushing strengths among the tested samples. At 14 days, strength continues to increase, albeit at a slower rate, with G1F3SC and G1F16SC still showing the highest strength, and G0F1SC continuing to exhibit the lowest strength. By 28 days, G1F3SC and G1F16SC show the highest strength, with G1F16SC reaching the peak, while G0F1SC remains the weakest. High-gypsum-content samples, such as G1F2SC and G1F3SC, continue to develop strength, whereas samples with lower gypsum content than G1F3SC experience a decrease in crushing strength, with noticeable strength retrogression in the later curing stages.

In Figure 6b, the strength distribution of the carbonized curing samples follows a distinct M-shaped trend across different curing periods. At 3 days, G1F4FHC and G1F16FHC exhibit relatively high strength, forming the peaks of the M-shape, with G1F4FHC showing the highest strength and G1F6FHC the lowest. At 7 and 14 days, G1F4FHC and G1F16FHC maintain high strength, continuing to form the peaks of the M-shape, with G1F16FHC showing the most significant increase in strength, while G1F6FHC remains the lowest. By 28 days, the strength continues to increase, with G1F16FHC achieving the highest strength and G0F1FHC showing the lowest. Unlike the decline observed in the SC samples, this demonstrates that FHC effectively prevents the issue of strength reduction over time. The control samples G0F1SC and G0F1FHC, which contain no gypsum, exhibit low early strength and slow strength growth. CFBFA alone, without gypsum, does not significantly enhance mechanical properties. The interaction between gypsum and CFBFA is crucial, as both G0F1SC and G0F1FHC consistently show the lowest strength from 7 to 28 days.

**Figure 7:**
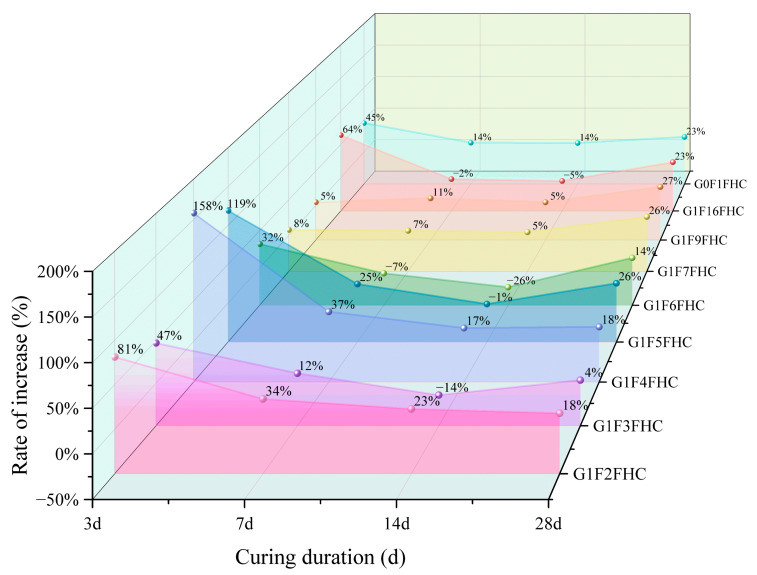
Comparison of the strength increase rate of FHC relative to SC at various curing ages.

As shown in Figure 7, most samples exhibit the highest growth rate at 3 days, indicating that FHC significantly enhances strength during the early curing stages. The strength of the samples subjected to FHC is consistently higher than that of samples with SC, primarily due to the synergistic effects of the residual heat and CO_2_ from the flue gas, which accelerate strength development. From 7 to 14 days, the growth rate decreases, likely due to the diminishing effects of carbonation and the increasing influence of hydration reactions. However, by 28 days, the growth rate rebounds, with strength continuing to improve in the FHC samples, while the SC samples experience a decrease in strength. This results in an overall increase in the growth rate, with the crushing strength growth rate at 28 days ranging from 4% to 27%, demonstrating that FHC continues to enhance crushing strength even at this later stage.

At 3 days, G1F4FHC shows the highest growth rate, indicating that pressurized flue gas heat curing is particularly beneficial for the crushing strength development of this sample. G1F4FHC exhibits a crushing strength 158% higher than G1F4SC, marking the highest growth rate among all samples. G1F6FHC, which shows the lowest strength, still demonstrates a 32% improvement over G1F6SC. The control sample, G0F1FHC, shows a 45% strength increase compared to G0F1SC, while G1F16FHC outperforms G1F16SC with a growth rate of 64%. At 28 days, G1F4FHC maintains the highest crushing strength, exceeding G1F4SC by 158%. G1F6FHC shows a 32% increase compared to G1F6SC. The control sample G0F1FHC shows a 45% increase over G0F1SC, and G1F16FHC achieves a crushing strength of 9.13 MPa, which is a 64% increase over G1F16SC.

The influence of gypsum content on strength growth is also evident. At 3 days, when the gypsum content is between 8.50% and 12.14%, the growth rate is lower than that of the gypsum-free control (G0F1FHC). However, when the gypsum content increases to between 14.16% and 28.30%, the growth rate exceeds that of the control group. This trend is attributed to the interaction between gypsum and CFBFA. As the gypsum content increases, the amount of CFBFA decreases, affecting their combined interaction. Gypsum catalyzes carbonation and promotes hydration reactions, enhancing strength. However, at lower gypsum contents (8.50–12.14%), the insufficient promotion of carbonation does not fully compensate for the reduction in CFBFA, which plays a crucial role in improving the microstructure and strength. As the gypsum content rises to between 14.16% and 28.30%, the promoting effect of gypsum becomes more pronounced, effectively accelerating carbonation and improving early strength, which compensates for the reduction in CFBFA, resulting in a higher growth rate compared to the gypsum-free control.

The influence of gypsum content on strength growth is also evident. At 3 days, when the gypsum content ranges from 8.50% to 12.14%, the increase rate is lower than that of the gypsum-free control (G0F1FHC). However, when the gypsum content increases to between 14.16% and 28.30%, the increase rate surpasses that of the control group.

The crushing strength of G1F16FHC increased by 69.31% compared to G0F1FHC, highlighting the significant role of gypsum in enhancing strength development. Furthermore, the crushing strength of G1F16FHC is 23.46% higher than that of G1F16SC, confirming the synergistic effect of gypsum addition and FHC in promoting strength development. The FHC process significantly accelerates early strength development.

In summary, the results indicate that FHC is a highly effective method for enhancing the strength of composite gravels, particularly during the early curing stages. The positive effects of FHC are further enhanced by gypsum content, and the balance between gypsum and CFBFA is crucial for achieving optimal strength.

#### 3.1.2. Apparent Density

**Figure 8:**
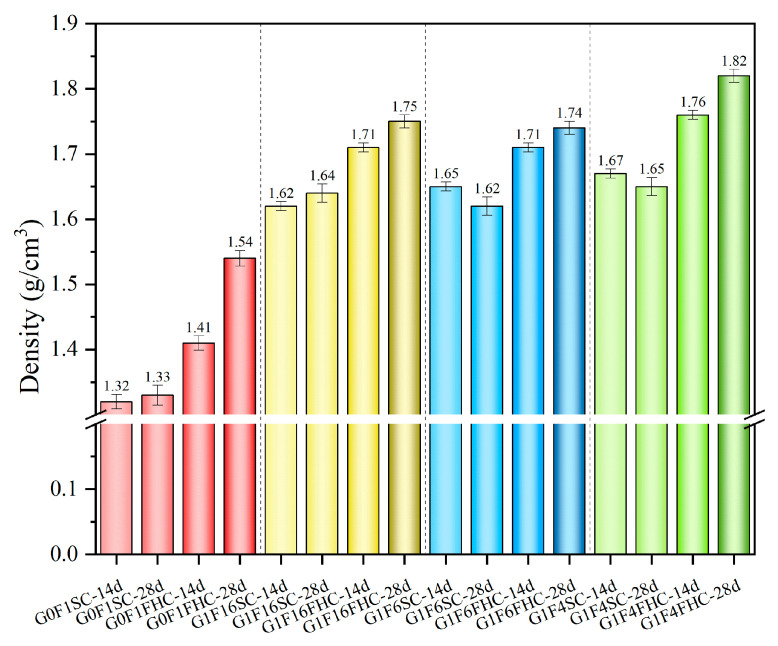
The apparent density of FHC and SC samples at different ages.

Figure 8 illustrates the evolution of apparent density in the composite gravels. It can be observed that the density increase in SC samples from 14 to 28 days is minimal, and even shows a slight decrease. In contrast, FHC samples exhibit a more consistent density increase. For every formulation and at both curing times, the FHC specimens show higher density than their SC counterparts, demonstrating that the CO_2_-heat curing regimen accelerates matrix consolidation, resulting in a consistently more compact composite gravel than standard curing.

#### 3.1.3. Water Absorption

**Figure 9:**
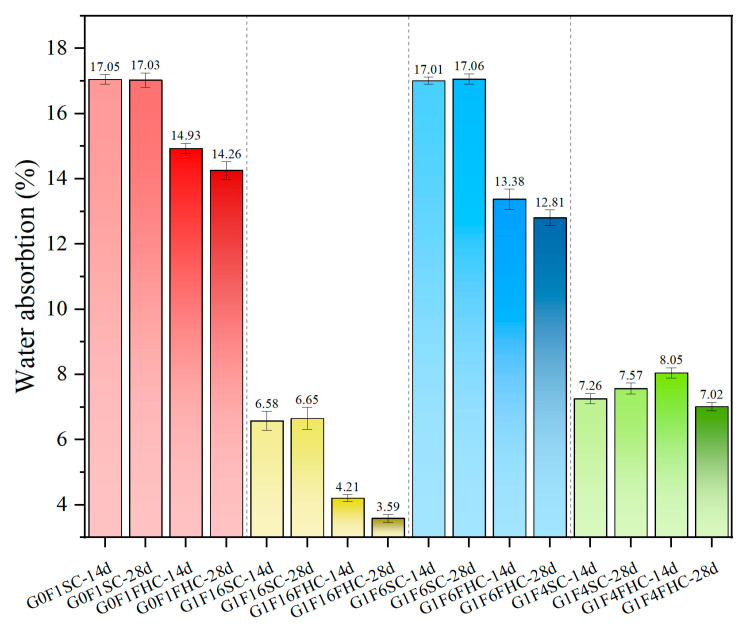
The 24 h water absorption of SC samples and FHC samples at different ages.

Figure 9 illustrates that FHC consistently reduces water absorption compared to SC. Notably, the water absorption of SC samples shows a slight increase from 14 to 28 days. In contrast, the FHC samples exhibit a clear reduction in water absorption over the same period. This trend highlights the positive impact of carbonation and elevated temperature in the FHC process, which results in a more compact structure and lower porosity.

Regarding the increase in water absorption between 14 and 28 days in G1F4SC, this sample belongs to the high-gypsum regime, where a substantial amount of unreacted gypsum remains even at later ages. While elevated gypsum dosage promotes early hydration and ettringite formation, excessive residual gypsum can trigger delayed hydration and expansion in later curing stages. This continued formation of expansive phases, such as needle-like ettringite, especially in localized areas, may result in microcracking and porosity rebound, thereby increasing capillary connectivity and water uptake. Furthermore, in SC samples with low gypsum content, the dissolution of anhydrite within CFBFA during late-stage curing induces internal expansion stress, thereby causing structural instability. In addition, under standard curing, the hydration environment lacks the CO_2_-induced pore refinement seen in carbonated systems. This makes the SC matrix more susceptible to sulfate-induced expansion without the compensating densification effect from CaCO_3_ precipitation. Therefore, the late-stage increase in water absorption is attributed to a combination of residual gypsum hydration, expansion-induced microcracking, and incomplete matrix densification.

### 3.2. Microstructure and Pore Structure

#### 3.2.1. SEM Analysis

**Figure 10:**
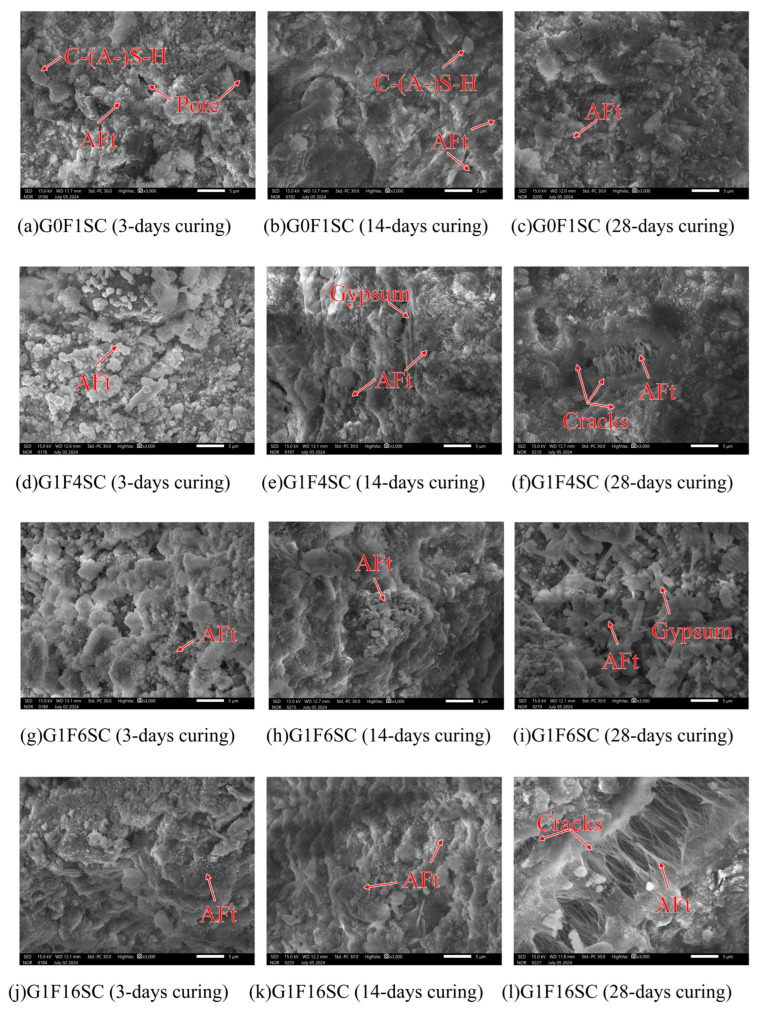
Representative SEM micrographs of standard curing composite gravels.

**Figure 11:**
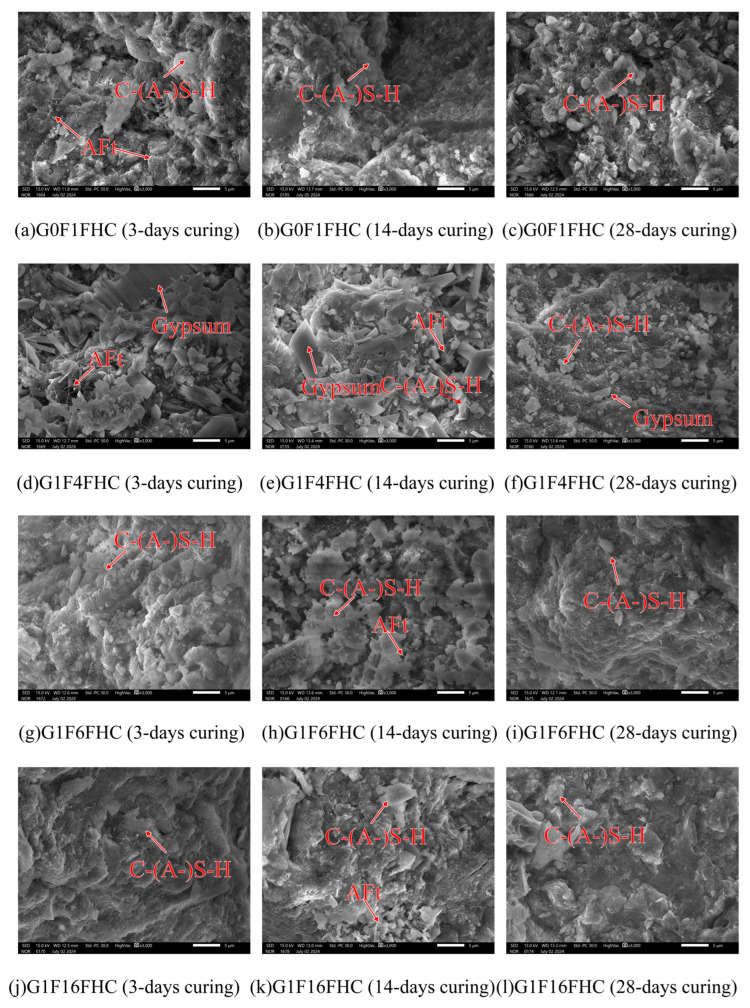
Representative SEM micrographs of pressurized flue gas heat curing composite gravels.

As shown in Figure 10 and Figure 11, the microstructural morphology of the composite gravels in the middle layer was observed under SEM at 3000× magnification. For a clear view of their macroscopic appearance and overall structure, please refer to Figure 20 in Section 4. This figure displays photographs of the same objects taken with a simple camera. The microstructure of composite gravels under SC and FHC conditions was analyzed using SEM at 3, 14, and 28 days. SEM characterization revealed progressive densification of the microstructure over time, with FHC-treated samples consistently demonstrating a denser and more compact structure compared to those under standard curing.

At 3 days, aggregated granular hydration products were observed in the G0F1SC sample, whereas the G0F1FHC sample exhibited only a few sporadic, elongated needle-like ettringite crystals [36]. Similar patterns were observed in the G1F4SC sample, which contained abundant aggregated granular hydration products, in contrast to the G1F4FHC sample, which exhibited sparse long needle-like ettringite crystals accompanied by significant unreacted gypsum. The G1F6SC sample contained a mixture of aggregated granular hydration products, while G1F6FHC showed sparse long needle-like ettringite crystals and substantial unreacted gypsum. In the G1F16SC sample, short needle-like ettringite was prevalent, but G1F16FHC displayed only C-(A-)S-H gel without visible ettringite.

By 14 days, a small amount of needle-like ettringite was observed in the G0F1SC sample, while G0F1FHC exhibited only a small amount of dispersed long needle-like ettringite. In G1F4SC, a gradual transformation of granular ettringite into needle-like crystals was noted, accompanied by substantial residual gypsum, indicating insufficient gypsum consumption. Conversely, the G1F4FHC sample still showed sparse needle-like ettringite along with unreacted gypsum. The G1F6SC sample contained clustered, robust ettringite, whereas G1F6FHC presented sparse long needle-like ettringite and unreacted gypsum. The G1F16SC sample showed needle-like ettringite crystals, while G1F16FHC displayed fewer, sporadic long needle-like ettringite crystals embedded within a dense matrix.

At 28 days, aggregated granular hydration products were still abundant in G0F1SC, but scarcely observed in G0F1FHC. In the G1F4SC sample, an excessive growth of delayed needle-like ettringite triggered internal expansion and cracking, significantly compromising the microstructural integrity. G1F4FHC continued to display substantial unreacted gypsum. Similarly, in G1F6SC, clustered needle-like ettringite crystals were observed, while G1F6FHC exhibited needle-like ettringite and significant unreacted gypsum. The G1F16SC sample experienced extensive internal expansion and cracking due to delayed needle-like ettringite formation, while the corresponding G1F16FHC sample presented a notably denser microstructure, underscoring the optimization provided by flue gas heat curing.

FHC significantly influenced the early morphology of ettringite, promoting the formation of needle-like structures due to accelerated reaction kinetics at elevated temperatures. Under FHC conditions, the needle-like ettringite morphology prevailed from early stages, with granular hydration products nearly absent. In contrast, under standard curing conditions, aggregated granular hydration products developed due to the higher-pH environment, consistent with the findings of previous studies, and XRD, FTIR, and thermogravimetric analysis (Section 3.3.1, Section 3.3.2 and Section 3.3.3) suggest they are ettringite [36,37]. According to prior research, at pH values of 13.0 and above, ettringite forms as granular aggregates. These undesirable granular ettringite aggregates can negatively impact both the expansion and mechanical properties of composite gravels. Granular ettringite lacks the structural integrity of needle-like ettringite, potentially leading to instability, excessive expansion, and a reduction in the strength and durability of the composite gravels [37]. In the FHC samples, the carbonation reaction lowered the overall system pH. These lower-pH conditions favored the formation of ettringite with a needle-like morphology rather than undesirable granular ettringite aggregates [38]. In SC samples, undesirable granular ettringite aggregates, initially formed at early stages, gradually transition into needle-like shapes by 28 days as hydration progresses. This transition can lead to internal expansion and microcracking, especially when delayed ettringite formation occurs excessively [39]. Compared to undesirable granular ettringite aggregates, the needle-like morphology formed during early hydration stages is more structurally advantageous [40]. Its elongated crystal shape more effectively fills pores, enhancing the internal cohesion and compactness of the composite matrix, thereby reducing the risk of detrimental expansion at later stages [41]. Consequently, samples cured under FHC conditions exhibited greater stability and less expansion at later ages compared to those cured under standard conditions.

#### 3.2.2. MIP Analysis

**Figure 12:**
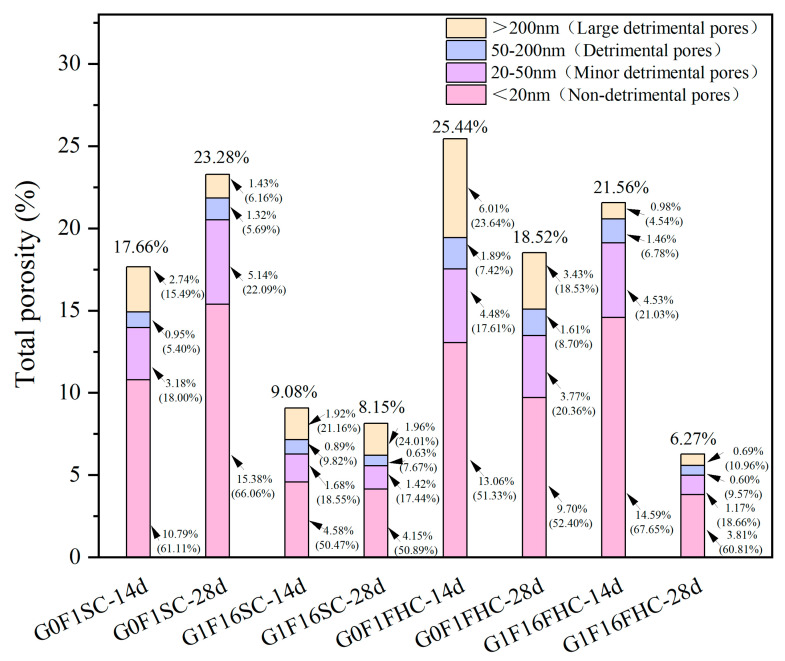
Total porosity and pore size distribution of G0F1SC, G0F1FHC, G1F16SC, and G1F16FHC samples.

Figure 12 shows the total porosity and pore size distribution of G0F1SC, G0F1FHC, G1F16SC, and G1F16FHC samples. In G0F1SC, total porosity grew from 17.66% to 23.28%. Although the fraction of large detrimental pores (>200 nm) almost halved (from 2.74% to 1.43%), the population of detrimental pores (50–200 nm) rose by roughly one-third (from 0.95% to 1.32%), and the 20–50 nm band almost doubled (from 3.18% to 5.14%). A comparable trend was observed for G1F16SC: the volume fraction of large detrimental pores stayed at roughly 2% between 14 and 28 days, yet the finer pore classes were scarcely reduced, so the overall porosity declined only marginally (from 9.08% to 8.15%). QXRD and SEM observations clearly reveal severe late-age degradation mechanisms under standard curing. In G0F1SC, ettringite and secondary gypsum crystallize preferentially within pre-existing capillary pores (>50 nm) and interfacial transition zones. Their expansive growth narrows some of the original >200 nm voids but simultaneously exerts pressure on the surrounding matrix, resulting in the formation of new defects in the 50–200 nm range. Consequently, while the fraction of large detrimental pores decreases, the proportion of detrimental pores increases, partly offsetting the apparent benefit of large detrimental pores refinement. In the G1F16SC mix, delayed ettringite formation (DEF) becomes dominant. Needle-like crystals undergo radial expansion, generating micro-cracks that propagate into the surrounding matrix. As a result, both 50–200 nm pores and >200 nm cracks increase slightly, and SEM images clearly reveal internal fissuring attributable to DEF. These pore-scale changes manifest at the macroscale: crushing strength declines, water absorption rises, and apparent density shows only a marginal increase, confirming that standard curing fails to refine the pore network—and can even exacerbate deterioration—in both G0F1 and G1F16 mixes. Under FHC, the harmful-pore spectrum contracts sharply. In G0F1FHC, total porosity decreases from 25.44% to 18.52%, driven by simultaneous reductions in both large detrimental pores (from 6.01% to 3.43%) and detrimental pores (from 1.89% to 1.61%). The effect is even more pronounced in G1F16FHC, where total porosity plummets from 21.56% to 6.27%, and the combined fraction of harmful pores drops below 1.3% at 28 days. The synergistic action of CO_2_ and elevated temperature promotes rapid early-age carbonation, leading to the precipitation of calcite and the formation of a dense microstructure. This early densification effectively blocks pore connectivity and suppresses the generation of new defects. The fraction of detrimental pores (50–200 nm) and large detrimental pores (>200 nm) also decreases, indicating that the harmful pore population is not replenished post-carbonation, and instead continues to decline through ongoing hydration. Large detrimental pores are governed chiefly by curing regime, while detrimental pores reflect an interplay between curing and mix design. Conventional SC fails to sustain pore refinement and can even provoke harmful-pore regeneration via delayed ettringite; by contrast, FHC delivers a sustained, multi-scale densification that underpins the superior strength, lower water uptake, and higher apparent density recorded for the FHC composites.

At both 14 d and 28 d, the G1F16 mix consistently exhibits a lower total porosity than its gypsum-free counterpart (G0F1) under both SC and FHC. Under SC, the reduced porosity of G1F16SC reflects the combined contribution of early ettringite formation and the gradual densification brought about by pozzolanic reactions. Under FHC, the same hierarchy persists—G1F16FHC remains denser than G0F1FHC. This superiority is evident not only in total porosity but also in the most critical pore classes: at 14 d, the fraction of large detrimental pores (>200 nm) is 0.98% in G1F16FHC versus 6.01% in G0F1FHC, and the 50–200 nm band is 1.46% versus 1.89%; similar gaps remain at 28 d (0.69% vs. 3.43% and 0.60% vs. 1.61%, respectively). The synergistic effect of rapid surface carbonation, early sulfate activation, and subsequent CFBFA reactions therefore promotes a more effective suppression of harmful pores in the G1F16 system. Overall, the initial pore infilling by early-formed ettringite, followed by the densifying action of later C–(A)–S–H gel growth, work in tandem to compact the microstructure—reducing both large (>200 nm) and medium (50–200 nm) detrimental pores—regardless of the curing route employed.

### 3.3. Carbonation Mechanisms and Phase Evolution

#### 3.3.1. CO_2_ Uptake

**Figure 13:**
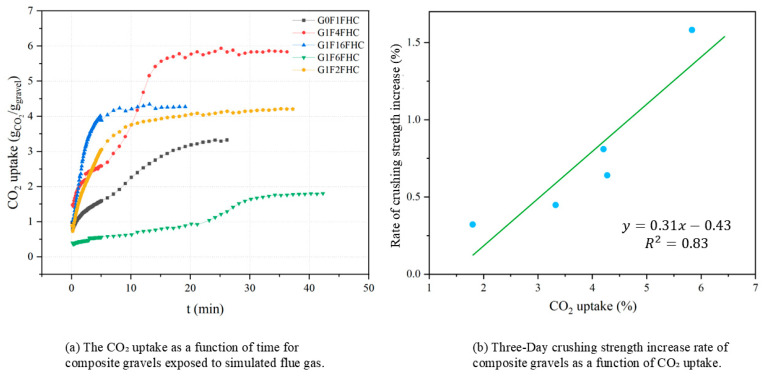
CO_2_ uptake and crushing strength increase in composite gravels exposed to pressurized flue gas.

Figure 13a shows the CO_2_ uptake of composite gravels during exposure to simulated flue gas at 1 MPa. The carbonation process is an irreversible heterogeneous reaction involving gaseous CO_2_, pore solution, and solid phases such as Ca(OH)_2_ or C–S–H [42]. G0F1FHC and G1F6FHC reach their maximum CO_2_ uptake within the first few minutes, after which the absorption curve flattens, and the rate of increase slows down, showing a relatively low absorption rate over time. In contrast, G1F4FHC and G1F16FHC exhibit relatively higher CO_2_ uptake, with a sustained high absorption rate for a longer period. Specifically, G1F4FHC shows the most significant increase in absorption within the first 15 min, indicating a stronger ability to absorb CO_2_.

During the carbonation process, calcium-rich phases such as silicates, Ca(OH)_2_, and ettringite undergo dissolution and/or reaction with CO_2_, which leads to the precipitation of CaCO_3_ from the pore solution. Under the experimental conditions, both the rate and extent of CO_2_ uptake are influenced by kinetic limitations, primarily related to the dissolution of CO_2_ in water and the subsequent carbonation reactions involving CFBFA solids [43]. These limitations are determined by the reactivity of cementitious material and the availability of reactive sites. The carbonation reaction occurs from the surface to the interior, with the surface reaction rate typically higher than that of the interior [44,45,46]. In the initial stage, the reaction proceeds rapidly, but as the reaction time increases, the rate gradually slows down. This phenomenon can be attributed to the following factors: (i) In the early stage of the reaction, the nucleation and growth rates of carbonate crystals are closely related to the surface area of the reactants. As the surface area increases, the reaction proceeds rapidly [47]. (ii) As the reaction progresses, the formed carbonate layer gradually thickens, and the microstructure becomes more compact, making it difficult for CO_2_ molecules to further diffuse to the reaction sites, thus limiting the reaction rate [48]. The process of carbonation between CO_2_ and the composite gravel proceeds as follows:

(4) $${CO}_{2}\left(g\right)+H_{2}O\left(l\right)↔H_{2}CO_{3}\left(aq\right)↔H^{+}\left(aq\right)+HCO_{3}^{-}\left(aq\right)$$

(5) $$HCO_{3}^{-}\left(aq\right)↔H^{+}\left(aq\right)+CO_{3}^{2-}\left(aq\right)$$

(6) $$Ca_{2}SiO_{4}\left(s\right)+4H^{+}\left(aq\right)\to 2{Ca}^{2+}\left(aq\right)+SiO_{2}\left(s\right)+2H_{2}O\left(l\right)$$

(7) $$2Ca_{3}SiO_{5}\left(s\right)+12H^{+}\left(aq\right)\to 6{Ca}^{2+}\left(aq\right)+2SiO_{2}\left(s\right)+6H_{2}O(l)$$

(8) $$Ca^{2+}\left(aq\right)+CO_{3}^{2-}\left(aq\right)\to CaCO_{3}\left(s\right)$$

(9) $$CaO\left(s\right)+H_{2}O\left(aq\right)\to Ca(OH{)}_{2}\left(s\right)$$

(10) $$Ca(OH{)}_{2}\left(s\right)+CO_{3}^{2-}\left(aq\right)+2H^{+}\left(aq\right)\to CaCO_{3}(s)+{2H}_{2}O\left(l\right)$$

(11) $$CaSO_{4}\left(s\right)+2H_{2}O\left(aq\right)\to CaSO_{4}\cdot 2H_{2}O\left(s\right)$$

(12) $$\begin{array}{l} Ca_{6}{Al}_{2}({SO}_{4}{)}_{3}(OH{)}_{12}\cdot {26H}_{2}O\left(s\right)+3CO_{3}^{2-}(aq)+{6H}^{+}(aq)3CaCO_{3}(s)+3(CaSO_{4}\cdot {2H}_{2}O)(s)+Al_{2}O_{3}\\ \;\;\;\;\;\;\;\;\;\;\;\;\;\;\;\;\;\;\;\;\;\;\;\;\;\;\;\;\;\;\cdot xH_{2}O(s)+{(29-x)H}_{2}O(l) \end{array}$$

(13) $$mCaO\cdot SiO_{2}\cdot nH_{2}O\left(s\right)+mCO_{3}^{2-}\left(aq\right)+2mH^{+}\left(aq\right)\to mCaCO_{3}\left(s\right)+SiO_{2}\left(s\right)+{\left(m+n\right)H}_{2}O\left(l\right)$$

From Equations (6), (7), (10), (12) and (13), it can be observed that the main carbonation raw materials include Ca_2_SiO_4_, Ca_3_SiO_5_, Ca(OH)_2_, ettringite, and C-S-H gel. The primary carbonation product is calcite (CaCO_3_). Among these, Ca_2_SiO_4_, Ca_3_SiO_5_, and Ca(OH)_2_ are also key raw materials in hydration reactions, classified as hydratable components. During the reaction between composite gravels and CO_2_, a series of complex chemical reactions occur. These reactions mainly include the dissolution of CO_2_ to form HCO_3_^−^, the dissolution of Ca-based materials, and the precipitation of calcite. In the carbonation process, not only is CO_2_ fixed, but calcite precipitates and by-products are also generated.

Among these, the G1F4FHC mixture shows the highest CO_2_ adsorption capacity, possibly due to the higher gypsum content enhancing the system’s reactivity, allowing more solid-phase calcium to participate in the reaction with CO_2_. According to Equations (6), (7), (10), (12), and (13), the carbonation reaction is a process that releases water while also causing material redistribution. Additionally, water is mainly released through the carbonation of Ca(OH)_2_ and changes in pore structure, while the carbonation of C-S-H gel releases significantly less water. Solid-phase calcium migrates from the C-S-H gel to the pore solution, where it reacts with CO_3_^2−^ to form CaCO_3_ [49]. The volume of carbonated C–S–H gel shrinks due to decalcification and silicate chain polymerization [50,51,52]. In contrast, the hydration of f-CaO and CaSO_4_ produces Ca(OH)_2_ and CaSO_4_·2H_2_O, respectively, both of which contribute to volume expansion, as shown in Equations (9) and (11). Therefore, utilizing the shrinkage effect of carbonation curing to compensate for expansion can effectively reduce porosity and improve structural stability. Furthermore, the carbonation of Ca(OH)_2_ produces CaCO_3_, which expands by approximately 11–14% [53]. Therefore, although the carbonation reaction is typically accompanied by some volumetric shrinkage, this shrinkage effect compensates for expansion, effectively reducing porosity and enhancing structural stability.

In a high-pressure CO_2_ environment, ettringite rapidly decomposes into CaCO_3_, gypsum, and aluminum gel [54]. The deposition of CaCO_3_ fills the pores, further refining the pore structure, and this structural optimization significantly enhances the mechanical properties of the material. CaCO_3_ primarily grows in the pores and on the surfaces of existing hydration products and aggregates [55]. This process highlights the dual contribution of CaCO_3_ to mechanical properties: it not only fills the pore structure but also strengthens the overall microstructure framework.

Figure 13b illustrates the correlation between CO_2_ uptake and the 3-day strength increase rate of FHC-treated samples relative to SC samples for various gypsum-to-CFBFA ratios. The data exhibit a linear relationship (y = 0.31x − 0.43, R^2^ = 0.83), indicating that greater CO_2_ uptake is directly associated with a higher rate of early-age strength gain under FHC conditions. This significant positive correlation underscores the crucial role of carbonation in enhancing the mechanical properties of composite gravels. Specifically, each unit increase in CO_2_ uptake corresponds to a strength increase rate increment of approximately 31% relative to standard curing [7,32,43]. These results demonstrate that carbonation not only facilitates CO_2_ sequestration but also substantially accelerates strength development, establishing FHC as an effective strategy for achieving both environmental sustainability and improved material performance. This finding is consistent with previous research demonstrating a positive relationship between CO_2_ uptake and early-age strength enhancement [32,56].

#### 3.3.2. XRD Analysis

**Figure 14:**
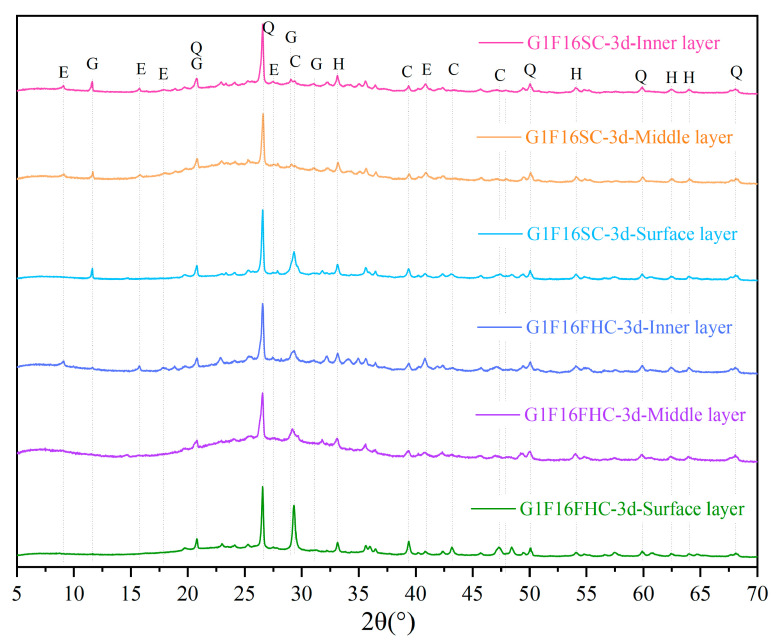
Comparison of XRD patterns of FHC and SC composite gravels at different locations (Q: quartz; E: ettringite; C: calcite; H: hematite; and G: gypsum).

Figure 14 presents a comparison of the layer-specific XRD patterns of 3-day-cured G1F16 specimens subjected to pressurized flue gas curing (FHC) and standard curing (SC). The FHC sample displays pronounced calcite peaks, whose intensity diminishes from the surface to the middle layer and is weakest in the core, confirming that carbonation produces the largest CaCO_3_ fraction at the exposed rim. No gypsum reflections are detected in any FHC layer, and ettringite appears only in the inner layer, indicating that most surface and middle layer AFt has reacted with CO_2_ to form calcite, thereby eliminating phases associated with delayed expansion.

In contrast, the SC specimen shows calcite only at the surface—attributable to limited atmospheric carbonation—while the middle and inner layers retain clear gypsum and ettringite peaks. The persistence of these sulfate phases, coupled with the absence of CaCO_3_ in the inner layer, points to an uncarbonated and potentially less stable microstructure. The XRD evidence demonstrates that pressurized flue gas curing converts expansive AFt into stable calcite, builds a dense outer shell, and establishes a beneficial surface-to-core carbonation gradient that enhances long-term durability.

**Figure 15:**
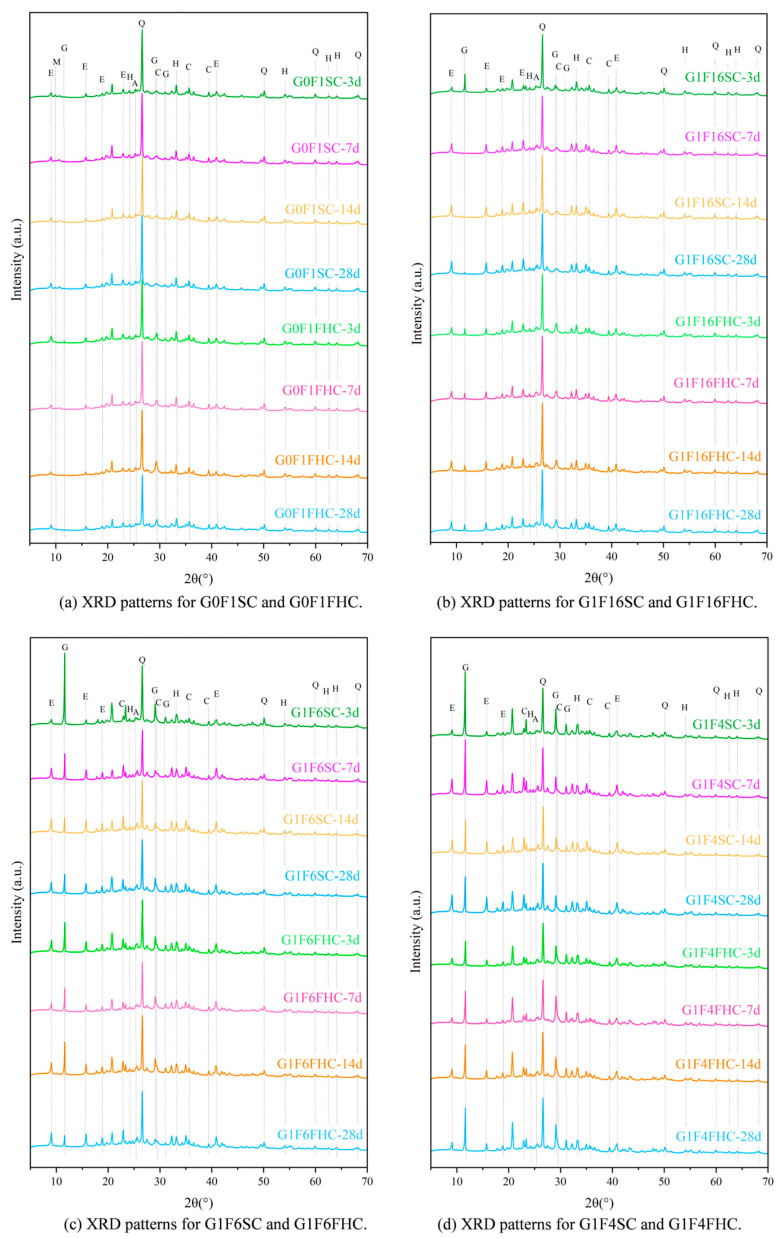
XRD patterns of 3 days, 7 days, 14 days, and 28 days. (Q: quartz; E: Ettringite; C: calcite; H: hematite; G: gypsum; and M: AFm; A: Anhydrite).

Figure 15 shows that the 3-day G0F1FHC paste already contains a weak gypsum peak, whereas no gypsum is detected in the corresponding G0F1SC sample. This peak results from the rapid conversion of anhydrite encapsulated in the CFBFA into gypsum. Since this transformation involves a 2.26-fold volume increase, releasing it early under FHC minimizes the subsequent cracking of the composite gravels. The immediate supply of gypsum, in turn, accelerates the synergistic hydration of CFBFA and Portland clinker, producing ettringite.

**Figure 16:**
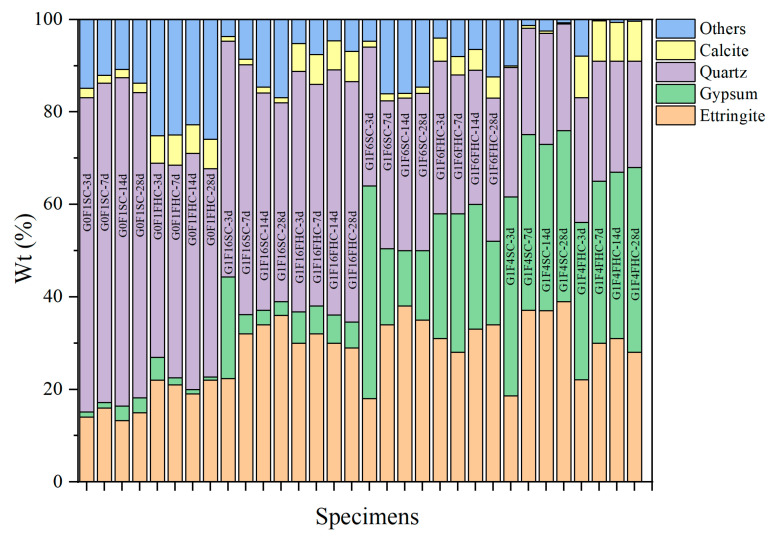
Quantitative mineral phase analysis of samples subjected to SC compared with those subjected to FHC curing.

Based on the analysis of crushing strength development and QXRD (Figure 16) results, SC samples with both high and low gypsum contents face potential strength degradation in the later stage. In low gypsum content samples, sulfate from gypsum is quickly consumed during the early stages of hydration, leading to the formation of ettringite. This accelerates early strength gain, explaining the initial rapid increase in strength at 3, 7, and 14 days. As hydration progresses beyond 14 days, sulfate from latent sources like anhydrite in the CFBFA begins to dissolve slowly. This delayed release of sulfate forms secondary ettringite within the hardened paste. This phenomenon, which resembles delayed ettringite formation (DEF), induces internal expansion, causing microcracks in the matrix. These late-forming ettringite crystals generate expansive stresses that damage the microstructure, leading to strength retrogression after 28 days. This accounts for the decline in strength observed in low gypsum samples at 28 days. As noted in the manuscript, the low gypsum samples, such as G1F16SC, show clear signs of gypsum consumption by day 3, with a subsequent dissolution of hard gypsum in CFBFA destabilizing the matrix and contributing to strength loss over time. In contrast, high gypsum content samples exhibit slower early strength development, but their strength continues to increase at 28 days. When the gypsum content is high, excessive gypsum may actually hinder the cement hydration process because it continues to react with the aluminates in the cement until all of the gypsum has been consumed. At this point, the excess gypsum not only affects the initial hydration rate of the cement, but it may also slow down the formation of some hydration products in the cement. Too much gypsum can result in more time being spent on consuming gypsum and generating ettringite, thereby delaying the early strength development of the cement. The higher gypsum content allows for sustained sulfate availability, promoting continuous ettringite formation and reducing the risk of premature microcracking and strength loss. However, high gypsum content samples, such as G1F2SC, may experience internal expansion and cracking at later ages due to excess gypsum, leading to potential risks, even though the strength does not decline at 28 days. High gypsum content in these mixes slows down early strength development, but because sulfate remains available throughout the hydration process, strength continues to improve in the long term. The risk, however, lies in the possibility of excessive expansion due to the continuous formation of ettringite. For samples with moderate gypsum content (e.g., G1F4SC), the gypsum content is neither too high nor too low. As a result, their cement hydration process does not proceed as slowly as that of high gypsum samples, nor does it consume sulfate too early, as in low gypsum samples. Therefore, these samples may experience a decline in strength and instability between 14 and 28 days. Quantitative XRD indicates that, although these samples are not as affected by cement retardation as the G1F2SC samples, they may still exhibit DEF phenomena, which results in expansion and microcracking due to the formation of secondary ettringite in the later stages. For samples such as G1F6SC, although DEF was not observed, the increase in gypsum content caused instability, which can still be attributed to the effect of anhydrite dissolved in the later stage transforming into gypsum.

Quantitative XRD (Figure 16) confirms that at 3 d the gypsum content in G1F16FHC is already much lower than in G1F16SC, indicating accelerated consumption under the pressurized flue gas regimen. From 7 d to 28 d, the rate of gypsum depletion in G1F16FHC slows, and a residual fraction remains at 28 d–a level that will be monitored to ensure long-term stability. By contrast, gypsum continues to increase in all SC mixes between 14 d and 28 d, showing that unreacted anhydrite is still dissolving and thus raising the risk of delayed expansion. A similar trend is observed for ettringite (AFt), except in the case of G1F6SC, where the AFt peak intensity shows a slight decline. In other SC mixes, the intensity of AFt increases steadily throughout the curing period. In contrast, FHC specimens exhibit their highest AFt intensity at 3 days, which then remains nearly unchanged over time.

Across all curing ages, the FHC samples exhibit stronger calcite peaks than their SC counterparts, demonstrating that FHC promotes rapid, deep carbonation. The SC specimens retain significant amounts of unreacted gypsum and freshly generated AFt after 28 d, whereas the FHC counterparts convert these potentially expansive phases into stable calcite within the first week. Together with the MIP evidence of fewer detrimental pores and the TG data showing higher CaCO_3_ contents, the XRD results demonstrate that pressurized flue gas heat curing not only accelerates early hydration but also suppresses harmful late-stage reactions, thereby delivering higher early strength and enhanced long-term stability.

#### 3.3.3. FTIR Analysis

**Figure 17:**
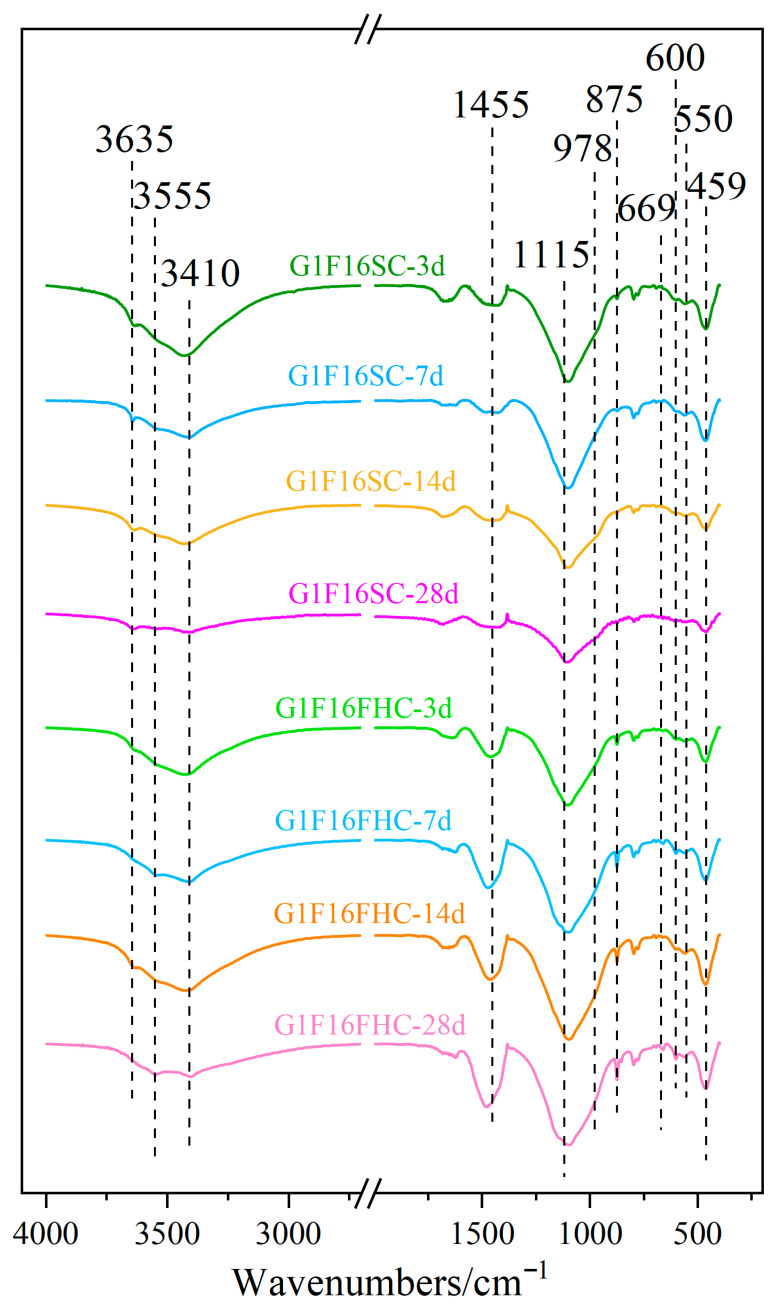
FTIR spectra of G1F16 at 3 days, 7 days, 14 days, and 28 days.

As shown in Figure 17, the strong band at 1455 cm^−1^ and the narrow band at 875 cm^−1^ correspond to the stretching vibrations of the C-O bond in carbonates [57]. The two crystallization water molecules in gypsum correspond to absorption peaks at 3410 cm^−1^ and 3555 cm^−1^, respectively. The absorption peak at 1115 cm^−1^ is attributed to the antisymmetric stretching vibration of sulfate, while the peaks at 669 cm^−1^ and 600 cm^−1^ correspond to the asymmetric bending vibrations of sulfate in dihydrate gypsum, and the peak at 459 cm^−1^ represents the symmetric bending vibration of sulfate in dihydrate gypsum. The experiments showed that at 3 days, the gypsum content in the SC samples was significantly higher than that in the FHC samples. As the curing period increased, Figure 16 indicated a gradual decrease in gypsum, but even by 28 days, a considerable amount of gypsum remained. The peak at 978 cm^−1^ corresponds to the asymmetric stretching vibration of Si-O-Si in the C-S-H gel [58]. The experimental results showed that the intensity of this absorption peak was slightly higher in the SC samples, indicating that the C-S-H gel content in these samples was higher than that in the FHC samples. This could be due to the reaction between CO_2_ and hydration products during the carbonation process, consuming part of the hydratable components and hydration products, leading to a reduction in the C-S-H gel content. The absorption peak at 3635 cm^−1^ represents the vibration of the structural OH^−^ groups in the hydration products. The experimental results showed that the intensity of this absorption peak was significantly higher in the SC samples, indicating that more hydration products were retained in these samples. During FHC, CO_2_ reacts with the hydration products, consuming some OH^−^ groups, resulting in a decrease in the intensity of this absorption peak. These findings are consistent with the XRD (Section 3.3.2) and TG-DTG (Section 3.3.3) analysis.

#### 3.3.4. TG-DTG Analysis

**Figure 18:**
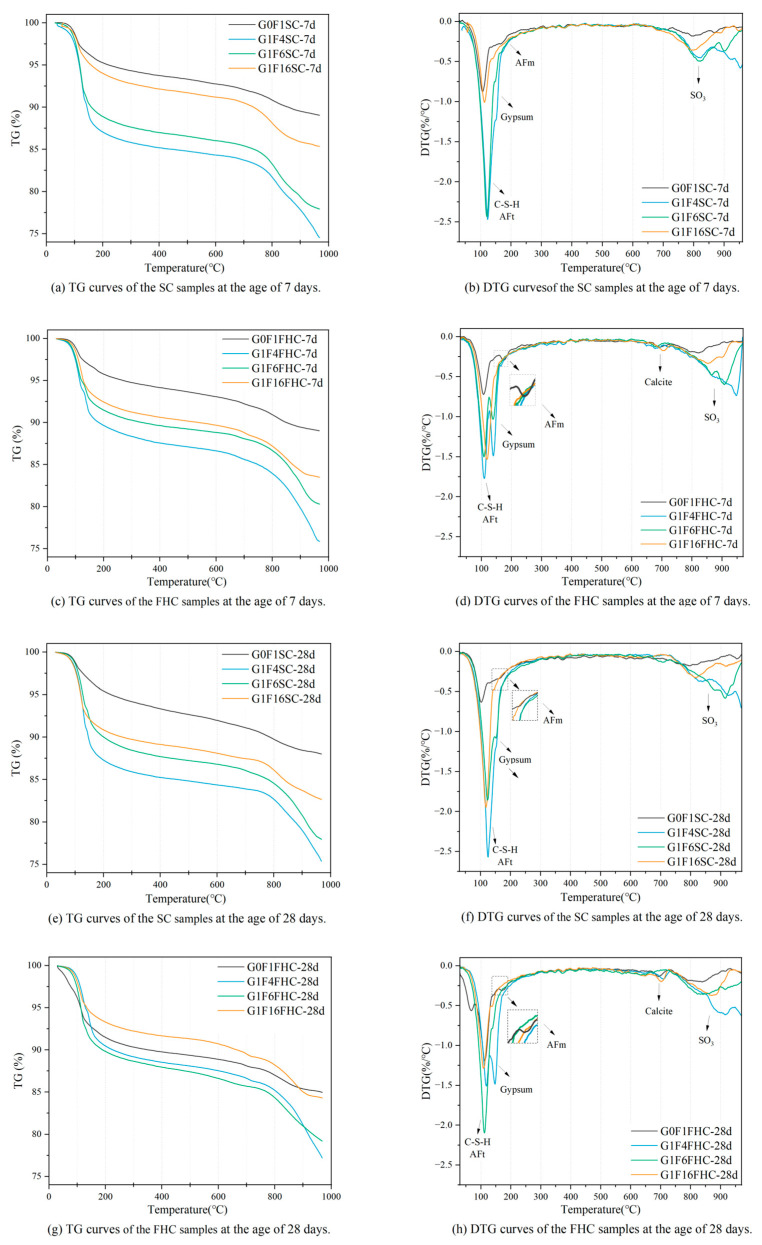
TG-DTG curves of SC and FHC composite gravels.

As shown in Figure 18, the first peak (30–128 °C) is primarily associated with the dehydration of hydration products, including ettringite and C-S-H gel [7]. The second peak (128–170 °C) mainly corresponds to the dehydration of gypsum [59]. The third peak (240–500 °C) corresponds to the loss of hydroxyl groups from Ca(OH)_2_ and amorphous C-S-H [60,61], while the fourth peak (500–760 °C) is attributed to the decarbonation of calcite [62]. A distinct peak of calcite can be observed in the FHC samples, whereas the peak of calcite is almost absent in the SC samples. Above 800 °C, mass loss is mainly caused by the release of SO_3_ from gypsum and CFBFA [7,32]. Based on the TG-DTG curve analysis, the generated amount of hydration products in samples under different curing conditions exhibits a clear regular trend. The mass loss of hydration products at 7 d for the FHC samples has the following order: G1F4FHC > G1F6FHC > G1F16FHC > G0F1FHC, while for FHC samples, the mass loss is as follows: G1F4SC > G1F6SC > G1F16SC > G0F1SC. This trend indicates that the formation of hydration products at 7 d increases substantially with the gypsum content. However, under FHC conditions, CO_2_ reacts with part of the hydration products and hydration components to form carbonated products, significantly reducing the amount of hydration products. The hydration product content of FHC samples is lower than that of SC samples. Regardless of the curing condition, increasing the gypsum content effectively promotes the generation of hydration products. This demonstrates that gypsum plays a significant role in enhancing hydration reactions and increasing the amount of hydration products.

The CO_2_ uptake followed the order G1F6FHC < G0F1FHC < G1F16FHC < G1F4FHC. Early carbonation preferentially consumed Ca(OH)_2_ and part of the aluminate phase, thereby lowering pore-solution pH and releasing bound water; the greater the CO_2_ uptake, the smaller the residual Ca(OH)_2_/Al(OH)_4_^−^ reservoir available for subsequent hydration. Consequently, the 28-day TG-DTG mass loss attributable to hydration products decreased in the sequence G1F6FHC > G0F1FHC > G1F16FHC > G1F4FHC. In particular, G1F4FHC, which absorbed the most CO_2_, lost almost all of its Ca(OH)_2_, while its residual SO_4_^2−^ maintained a high pH and stabilized AFt; this combination suppressed both further hydration and CaCO_3_ densification, giving the lowest hydration-product content. These findings indicate that CO_2_ thermal curing applied in FHC samples has a “double-edged” effect: moderate CaCO_3_ precipitation refines the pore structure, whereas excessive Ca(OH)_2_ consumption hinders later hydration.

In the gypsum-free samples G0F1SC and G0F1FHC, the AFm phase was detected, whereas no such phase was observed in the gypsum-containing samples. This is consistent with what is detected in XRD. Gypsum is the principal source of sulfate in the system; the abundant SO_4_^2−^ drives the aluminates to precipitate preferentially as ettringite, thereby suppressing AFm formation. Once the dissolved sulfate is exhausted, the AFt gradually dissolves and transforms into AFm. Consequently, the higher sulfate concentration maintained by added gypsum increases the stability of AFt and markedly retards its subsequent conversion to AFm [63,64].

Between the two gypsum-free pastes, the AFm content in G0F1FHC is higher than that in G0F1SC because flue gas thermal curing supplies additional CO_2_ and heat, jointly lowering the pore-solution pH and accelerating the AFt-to-AFm transformation. The lower pH promotes the rapid nucleation and stacking of plate-like AFm crystals, leading to potential instability and reduced durability [65,66]. Therefore, samples lacking the addition of gypsum are not suitable for FHC.

### 3.4. Mechanisms Behind Gypsum Addition and FHC Effects on Composite Gravels

The above findings demonstrate that FHC offers substantial potential for improving the stability of CFBFA-based composite gravels. This enhancement can be attributed to the following mechanistic pathways:

Regarding the increase in crushing strength, as the carbonation and hydration reactions progressed, the hydratable components were gradually consumed. The carbonation and hydration reactions compete for hydratable components. Gypsum plays a dual role by promoting the hydration of CFBFA and catalyzing the carbonation process, achieving a balance between the two reactions when the gypsum-to-CFBFA ratio is 1:16. Furthermore, a denser microstructure and improved mechanical properties were achieved when the carbonation and hydration reactions reached an optimal balance. This microstructure created a favorable internal environment for FHC, promoting the formation of crystalline phases such as ettringite and calcite. These crystalline phases, believed to have higher hardness than C-S-H gels, contribute to the improvement in crushing strength [67,68,69,70].

At early hydration stages, the mass loss assigned to hydration products rises with gypsum dosage in both curing modes, but is consistently lower under FHC because CO_2_ converts part of the hydrates to carbonates. CO_2_-uptake data indicate that excessive carbonation consumes most Ca(OH)_2_ and Al phases, curbing further hydration and CaCO_3_ densification; moderate carbonation refines pores without exhausting the alkaline reserve, giving the best balance. Phase analysis shows that gypsum suppresses AFm by sustaining a high SO_4_^2−^ level that stabilizes AFt; once sulfate is depleted, AFt dissolves and converts to AFm. Gypsum-free pastes therefore form more AFm, especially under FHC where CO_2_ and heat lower pH and accelerate the AFt-to-AFm conversion, producing plate-like AFm that can compromise long-term durability. Hence, an optimized gypsum/CFBFA ratio (1:16) and controlled carbonation are essential: sufficient gypsum promotes hydration and limits AFm formation, while moderate FHC densifies the matrix through benign CaCO_3_ precipitation without depleting Ca(OH)_2_. Samples lacking gypsum are unsuitable for FHC because the accelerated AFt-to-AFm conversion undermines stability.

Under SC, ettringite initially forms as undesirable granular ettringite aggregates that lack the structural integrity of rod-like crystals, leaving the matrix vulnerable to instability and excessive expansion. FHC suppresses this granular growth, promoting dense, needle-shaped ettringite instead and thereby preventing the expansion-related damage observed in SC specimens. The rod-like ettringite crystals formed under FHC interlace with C-(A-)S-H gel, improving the material’s cohesion and density.

FHC alters the hydration process by consuming hydration products and promoting calcite formation, which compensates for the loss of hydration products. Although FHC results in fewer hydration products and reactive components overall, it significantly increases the calcite peaks in the carbonated samples, indicating the rapid formation of calcite during carbonation. CaCO_3_ primarily forms in the pores and on the surfaces of existing hydration products, with calcium migrating from the C-S-H gel into the capillary pores [29]. This redistribution of materials occurs as calcium dissolves into the pore solution and reacts with CO_3_^2−^ to form CaCO_3_, refining the pore structure [24,44]. This sequence of reactions markedly improves the composite gravel’s stability. Under FHC, the volumetric shrinkage produced by carbonation offsets the expansion associated with hydration, thereby lowering porosity and reinforcing the microstructure. Moisture loss is driven mainly by the carbonation of Ca(OH)_2_ and pore closure; C–S–H contributes only marginally. The carbonation-induced decalcification of C–S–H causes slight [70,71] contraction [50,51,52], while the hydration of f-CaO and CaSO_4_ forms Ca(OH)_2_ and CaSO_4_·2H_2_O, both expansive. The carbonation of Ca(OH)_2_ itself yields CaCO_3_ with an 11–14% volume increase, counter-balancing the expansion generated by ongoing hydration in the CFBFA matrix. This process leads to a refined pore structure, a substantial reduction in large-scale capillaries, and consequently, enhanced crushing strength

FHC effectively reduces porosity and promotes the densification of the composite gravel’s microstructure. This process significantly enhances the crushing strength of the composite gravel by decreasing the proportion of large capillaries and optimizing the pore structure. Therefore, utilizing the shrinkage effect of FHC to compensate for expansion can effectively reduce porosity and improve structural stability.

Pressurized CO_2_ rapidly converts surface and mid-depth carbonatable components into calcite, creating a dense, carbonated rim while eliminating the expansive sulfate phases that persist in SC specimens. Early CaCO_3_ precipitation, together with accelerated hydration, fills large capillaries, driving a sustained reduction in harmful pore volume. In contrast, SC counterparts develop coarser pores as late-forming ettringite and gypsum disrupt the matrix. CO_2_ uptake correlates linearly with the early-age increase rate of crushing strength, demonstrating that carbonation contributes directly to mechanical densification. Thus, FHC establishes a beneficial surface-to-core carbonation gradient, refines the pore network, and locks CO_2_ into stable calcite—jointly delivering higher strength, lower water absorption, and enhanced long-term dimensional stability.

In SC samples, delayed ettringite growth during later hydration leads to the excessive formation of ettringite, causing microcracking and damaging the microstructure. By accelerating the early consumption of gypsum and promoting the conversion of anhydrite in CFBFA to gypsum during early hydration, excessive volumetric expansion is prevented. This process promotes early hydration and ettringite formation, which enhances early strength and helps prevent any decline in crushing strength. The early release of the expansion source enhances pore reduction, allowing the porosity to continue decreasing between 14 and 28 days, thereby improving stability. Although only a small amount of residual gypsum remains in the FHC samples at 28 days—indicating that gypsum hydration is not yet fully complete—this residue could still trigger expansion problems over a longer service life, particularly under high-humidity or cyclic wet–dry conditions. Future work should therefore focus on optimizing FHC parameters to promote a more complete gypsum reaction, or on minimizing expansion risk by carefully adjusting the gypsum dosage. Overall, while the residual gypsum at 28 days has little effect on short-term performance, its potential impact on long-term stability must be acknowledged and assessed through ongoing monitoring.

### 3.5. Results Comparison, Scientific Contribution, and Future Outlook

The findings from this study highlight the significant impact of pressurized flue gas heat curing (FHC) on the mechanical and microstructural properties of composite gravels, particularly those incorporating gypsum. Notably, the synergistic effects of gypsum and carbonation curing are evident.

The 28-day performance of the present CFBFA-based composite aggregate clearly surpasses or matches the best values reported in the recent literature. Xu et al. [72] obtained 9.01 MPa for a gypsum–hydrated-lime–activated CFBFA aggregate cured by flue gas carbonation, whereas our material reaches 9.13 MPa by the same age, highlighting the beneficial synergy between the tailored gypsum/CFBFA ratio and the pressurized FHC regimen. Pore-structure data further underline this advantage: Doleželová et al. [73] observed that the total porosity of gypsum-based composites increases after high-temperature exposure, but the present FHC-treated aggregate shows a marked porosity reduction, indicating that early CO_2_-induced CaCO_3_ precipitation effectively blocks large capillary voids and suppresses later microcracking. Water absorption is likewise competitive. Zhu et al. [74] reported that CO_2_-cured recycled coarse aggregates still exhibited higher absorption than the 3.59% recorded for our product, despite the beneficial influence of carbonation in their study. Taken together, the combination of higher crushing strength, denser pore network, and lower water uptake demonstrates the technical superiority of the FHC-activated CFBFA composite gravels and confirms its potential as a next-generation low-carbon lightweight aggregate for structural and durability-critical applications.

The scientific novelty of this study lies in the innovative use of pressurized flue gas heat curing (FHC) combined with gypsum to enhance the mechanical and microstructural properties of composite gravels, particularly those incorporating CFBFA. This research identifies the optimal gypsum-to-CFBFA ratio (1:16) that results in superior early-stage strength development, a key factor in improving the long-term performance of composite gravels. This study reveals that the synergy between gypsum and carbonation curing not only accelerates the early hydration process but also promotes a dense microstructure, contributing to enhanced durability and stability. The resulting material meets the Technical Guidelines for Construction of Highway Roadbases (JTG/TF20-2015) and thus presents a scalable low-carbon alternative to natural gravel, valorizing both CFBFA and flue gas CO_2_. These findings have significant applied value, particularly in the development of sustainable, low-carbon construction materials that can reduce environmental impact while improving performance in construction applications.

However, the applicability of these results is subject to certain limitations. First, the study focused on specific curing conditions, namely 80 °C, 1 MPa, and 15% CO_2_, which may not fully represent the range of environmental conditions encountered in real-world applications. Furthermore, while the study demonstrates substantial improvements in strength and durability, the long-term performance of the materials under diverse environmental stresses, such as freeze–thaw cycles or sulfate attacks, requires further investigation. Additionally, the potential environmental impacts of using gypsum and CFBFA in large-scale applications remain to be evaluated in greater detail.

Looking forward, future research should focus on exploring the effects of varying curing conditions, including different CO_2_ concentrations, temperatures, and pressures, on the properties of composite gravels. Long-term durability testing, particularly under aggressive environmental conditions, will be crucial in determining the practical applicability of these materials in real-world construction projects. This holistic approach to material optimization ensures that the use of FHC for composite gravels not only advances strength and durability but also contributes to sustainable development in the construction industry.

## 4. Life Cycle Assessment of CFBFA-Based Composite Gravels

**Figure 19:**
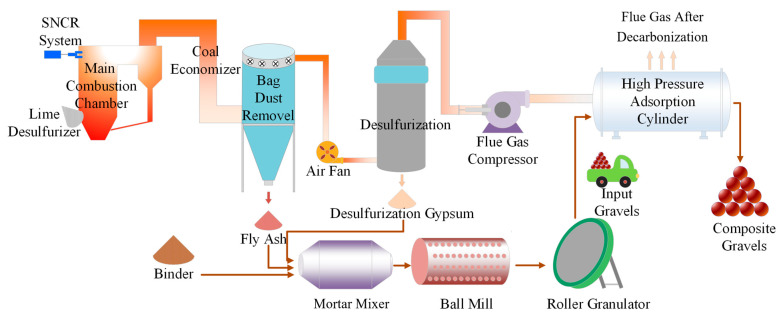
Composite gravel production line based on power plant infrastructure.

The FHC technology, with its unique mineral carbonation bonding mechanism, is gradually replacing traditional high-energy steam curing processes, heralding a green revolution in the construction industry. Driven by this innovative technology, LCA has become a vital tool for evaluating the environmental impacts of construction projects. LCA precisely quantifies the overall environmental impact by analyzing the entire life cycle of building materials, from raw material acquisition to final product formation. The composite gravel production line based on power plant infrastructure is shown in Figure 19. The primary raw materials include Portland cement, hydrated lime, desulfurized gypsum, and CFBFA. The amount of desulfurized gypsum was corrected for its purity to match the active ingredient content of analytical grade gypsum in all formulations. After transportation to the production site, these raw materials undergo mixing, stirring, and ball milling processes sequentially. The mixed materials are then processed into granular form through a pelletizing process, laying the foundation for the subsequent carbonation curing stage. During the carbonation curing phase, flue gas is injected into a high-pressure tank to mineralize the composite gravels, significantly enhancing the material’s mechanical properties and stability. Finally, the composite gravels undergo further curing under standard conditions to ensure they meet performance requirements.

**Figure 20:**
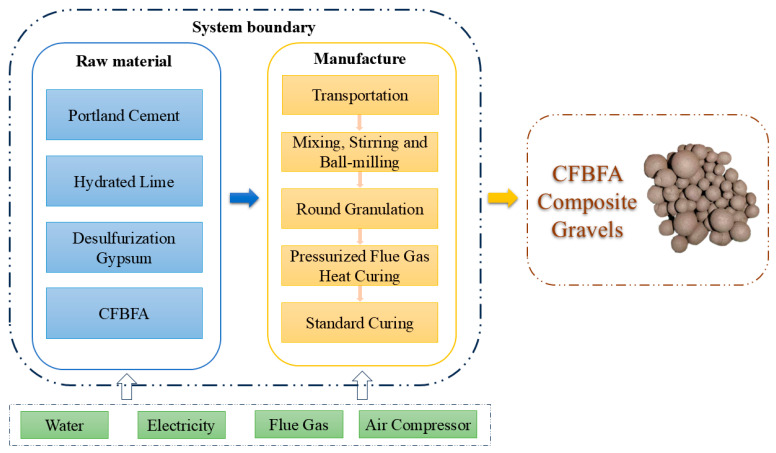
Life Cycle Assessment system boundary of FHC CFBFA-based composite gravels.

As shown in the Figure 20, the life cycle flowchart of CFBFA-based composite gravel illustrates the complete production process. The calculation boundary from cradle to gate has been defined, considering all processes from raw material acquisition to product shipment, including material and energy usage. The efficient use of auxiliary resources is critical throughout the production process, including water, electricity, flue gas, and air compressors, which are used for mixing, equipment operation, carbon dioxide gas supply, and gas compression, respectively. In this study, we focused on two key environmental impact indicators—GWP and CED. The results demonstrate that carbonation mineralization curing technology exhibits great potential in reducing greenhouse gas emissions and conserving energy, providing strong support for the green development of the construction industry.

(14) $$I_{i}={IR}_{i}+{IP}_{i}{+IT}_{i}$$

(15) $${IR}_{i}=\sum_{j=1}^{n}\left(W_{j}\times {IP}_{j,i}\right)$$

(16) $${IP}_{i}={IP}_{mix,i}+{IP}_{milling,i}+{IP}_{rolling,i}+{IP}_{CC,i}$$

(17) $${IP}_{GWP}=\left(\sum_{K=1}^{n}\left({IP}_{k,\;\;\;GWP}\right)\right)-C$$

(18) $${IT}_{i}=\sum_{j=1}^{n}\left(W_{j}\times D_{j}\times {IT}_{j,i}\right)$$

The value denoted as $I_{i}$ corresponds to the indicator for the *i*-th type of environmental impact assessment. The term $I_{GWP}$ signifies the assessment value for the potential contribution to global warming. The data labeled as ${IR}_{i}$ pertain to the evaluation of impacts at the stage of raw material acquisition, while ${IP}_{i}$ and ${IT}_{i}$ refer to the assessments of effects during the manufacturing process and transportation stages, respectively. The variable $W_{j}$ indicates the mass per unit of the *j*-th category of raw materials. The expression ${IP}_{j,i}$ denotes the unit mass values of the initial j categories of raw materials that relate to the i-th class of impact indicators. The terms ${IP}_{mix,i}$, ${IP}_{milling,i},{IP}_{rolling,i}$, and ${IP}_{CC,i}$ signify the respective amounts of energy consumed during the processes of mixing, grinding, rolling, and mineralization. In the context of the GWP index, the calculation of ${IP}_{GWP}$ follows the methodology outlined in Formula (10). The symbol *C* represents the quantity of carbon sequestered per unit of mass in the CFBFA-based composite gravel. The data on greenhouse gas emissions and energy consumption for raw materials are sourced from actual operational data and relevant studies [18,70,71,75,76].

**Figure 21:**
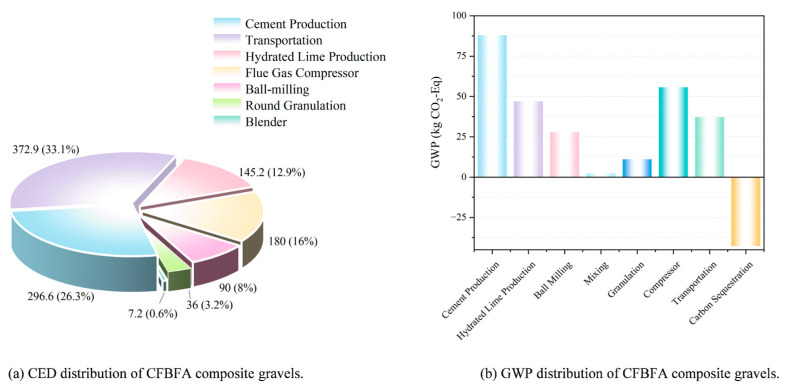
CED and GWP distribution of CFBFA composite gravels.

Figure 21a illustrates that the cumulative energy demand (CED) of the product is relatively low. The energy consumption for the composite gravels in this study was approximately 1127.88 MJ per ton. The production of CFBFA composite gravel takes place at a power plant, which minimizes transportation-related energy consumption. Both CFBFA and desulfurized gypsum can be utilized directly at the power plant, eliminating the need for transportation. Only cement and hydrated lime require transportation, with the distance calculated based on a 100 km truck transport from the cement plant to the production facility. As shown in the Figure 21b, the GWP distribution of the samples reveals that cement and hydrated lime production are the primary contributors to GWP, followed by transportation and mixing processes. The CO_2_ emissions from these stages significantly impact global warming. However, the adoption of FHC technology has significantly decreased the GWP, achieving a value of 226.38 kg CO_2_-Eq per ton. This technology reduces CO_2_ emissions by sequestering CO_2_ within the material, in contrast to traditional standard curing methods. Additionally, the production of CFBFA composite gravel at power plants, by utilizing waste materials such as CFBFA, desulfurization gypsum, and flue gas, minimizes the need for extra transportation and processing, thereby further decreasing the overall GWP.

## 5. Conclusions

Based on pressurized flue gas heat curing (FHC), this study proposes a novel approach for the resource utilization of CFBFA in conjunction with other industrial solid wastes, flue gas, and waste heat, to prepare high-performance CFBFA-based composite gravels. The mechanistic study supports the following conclusions:

(1) The optimal preparation of CFBFA-based composite gravels using pressurized flue gas heat curing (FHC) was achieved at a gypsum-to-CFBFA ratio of 1:16, yielding a 28-day crushing strength of 9.13 MPa, a CO_2_ uptake rate of 4.27%, an apparent density of 1.75 g/cm^3^, and a water absorption rate of 3.59%.
(2) FHC samples exhibited increased crushing strength, higher apparent density, lower water absorption, and a progressively denser microstructure over time, without the stability deterioration observed in SC samples. These results clearly demonstrate that FHC substantially improves the stability of CFBFA-based composite gravels by refining the pore structure, stabilizing expansive phases, and enhancing long-term durability—thereby effectively addressing the inherent challenges of CFBFA utilization.
(3) FHC effectively mitigates potential expansion in CFBFA-based composite gravels by rapidly consuming expansive sources such as gypsum and ettringite during the early curing stages. The accelerated hydration and carbonation reactions facilitated by FHC promote the early formation and subsequent stabilization of these phases, thereby preventing their excessive accumulation and late-stage expansion.
(4) An optimized gypsum/CFBFA ratio (1:16) provides sufficient sulfate to catalyze carbonation while preventing excessive carbonation, which is essential for ensuring matrix stability. Gypsum-free systems are prone to instability under FHC due to rapid AFt-to-AFm conversion and a lack of sulfate needed to stabilize ettringite. Excessive carbonation can deplete the alkaline reserve, hindering further matrix densification and long-term durability.
(5) Under SC, ettringite initially forms as undesirable granular ettringite aggregates that lack the structural integrity of rod-like crystals, leaving the matrix vulnerable to instability and excessive expansion. FHC suppresses this granular growth, promoting dense, needle-shaped ettringite instead and thereby preventing the expansion-related damage observed in SC specimens.
(6) A multi-dimensional assessment demonstrates that FHC induces a pronounced carbonation gradient in CFBFA-based composite gravels, converting expansive ettringite at the rim into stable calcite and forming a dense, durable outer shell. Consequently, under FHC conditions, CO_2_ uptake is significantly and positively correlated with the early-age crushing strength increase rate compared to standard curing.
(7) The adoption of FHC technology enabled the production of CFBFA composite gravels with a low cumulative energy demand (1127.88 MJ per ton) and a significantly reduced global warming potential (226.38 kg CO_2_-eq per ton). This reduction is achieved by sequestering CO_2_ within the material and utilizing solid waste resources directly at the power plant, thereby minimizing additional transportation and processing emissions.

## Figures and Tables

**Table 1:** Physicochemical properties of materials.

Material	CFBFA	Portland Cement	Gypsum	Hydrated Lime
Chemical composition				
(wt%)				
SO_3_	3.075	4.029	51.549	0.647
CaO	5.067	53.678	43.205	93.886
SiO_2_	46.237	21.245	-	0.928
Al_2_O_3_	29.502	7.298	0.794	0.956
Fe_2_O_3_	9.875	4.591	-	-
MgO	-	6.037	3.925	2.511
Physical properties				
Specific surface area (m^2^/kg)	333.6	213.0	84.95	974.5
D10 (µm)	2.11	6.14	15.65	1.93
D50 (µm)	16.98	21.89	49.66	9.66
D90 (µm)	48.37	50.46	111.7	26.04

**Table 2:** Design of CFBFA-based composite gravel feedstock ratios under different curing conditions.

Sample	Cement/wt%	Hydrated Lime/wt%	Gypsum/wt%	CFBFA/wt%
G0F1	10	5	0	85
G1F2	10	5	28.3	56.7
G1F3	10	5	21.25	63.75
G1F4	10	5	17	68
G1F5	10	5	14.16	70.83
G1F6	10	5	12.14	72.86
G1F7	10	5	10.63	74.38
G1F9	10	5	8.5	76.5
G1F16	10	5	5	80

## Data Availability

The original contributions presented in this study are included in the article. Further inquiries can be directed to the corresponding author.

## References

[1] Mei N., Zhi W., Jue-Shi Q., Sheng-Xuan T. (2019). Characteristics of Fluidized Bed Coal Combustion Fly Ash and Slag and Its Adaptability with Current Standards. Bull. Chin. Ceram. Soc..

[2] Li X., Chen Q., Huang K., Ma B., Wu B. (2012). Cementitious properties and hydration mechanism of circulating fluidized bed combustion (CFBC) desulfurization ashes. Constr. Build. Mater..

[3] Nguyen H.-A., Chang T.-P., Shih J.-Y., Chen C.-T., Nguyen T.-D. (2015). Influence of circulating fluidized bed combustion (CFBC) fly ash on properties of modified high volume low calcium fly ash (HVFA) cement paste. Constr. Build. Mater..

[4] Park S.M., Lee N.K., Lee H.K. (2017). Circulating fluidized bed combustion ash as controlled low-strength material (CLSM) by alkaline activation. Constr. Build. Mater..

[5] Liu X., Li Y., Zhang L., Cang D. (2010). Utilization of CFB fly ash in Eco-cement: Mechanical properties and microstructural analysis. Adv. Mater. Res..

[6] Chen G.-Y., Huang W.-H. (2021). Activation of Blast Furnace Slag with CFB Fly Ash as a Supplementary Binder Material: Hydration Products and Effects of Sulfate Attack. Crystals.

[7] Ren K., Ma S., Feng Y., Xu N., Bai S. (2023). Study on the composite gravel preparation and the synergistic absorption of CO_2_ by fly ash of CFB boiler. Fuel.

[8] Liu W., Liu X., Zhang L., Wan Y., Li H., Jiao X. (2024). Rheology, mechanics, microstructure and durability of low-carbon cementitious materials based on circulating fluidized bed fly ash: A comprehensive review. Constr. Build. Mater..

[9] Jia G., Wang Y., Yang F. (2022). A Review on the Application of Circulating Fluidized Bed Fly Ash in Building Materials. Adv. Mater. Sci. Eng..

[10] Ohenoja K., Pesonen J., Yliniemi J., Illikainen M. (2020). Utilization of Fly Ashes from Fluidized Bed Combustion: A Review. Sustainability.

[11] Zhang W., Liu X., Zhang Z., Li Y., Gu J. (2022). Synergic effects of circulating fluidized bed fly ash-red mud-blast furnace slag in green cementitious materials: Hydration products and environmental performance. J. Build. Eng..

[12] Wu C.-R., Zhan B.-J., Hong Z.-Q., Cui S.-C., Cui P., Kou S.-C. (2022). Hydration behavior of circulating fluidized bed fly ash (CFBFA) as a cementitious binder. Constr. Build. Mater..

[13] Chen Q., Lv G., Jiang X., Zhao X., Kong L. (2019). Stabilization of heavy metals in municipal solid waste circulating fluidized bed incineration fly ash by fusion–hydrothermal method. Waste Dispos. Sustain. Energy.

[14] Kumar S., Murthi P., Awoyera P., Gobinath R., Kumar S. (2022). Impact Resistance and Strength Development of Fly Ash Based Self-compacting Concrete. Silicon.

[15] Liang D.-C., Yang G.-M., Zhang X.-L., Liu D.-Q., Liu J.-C., Xie Q. (2021). Synthesis of Geopolymer from Fly Ash and Optimization of Process Parameters by Neural Network. J. China Coal Soc..

[16] Cheng Y., Wang X., Chen J., Yu H., Shen J., Luo X., Li J. (2023). The performance of bubble-mixed lightweight soil to relieve expansion of circulating fluidized-bed fly ash. J. Build. Eng..

[17] Zhang L., Su X., Zhang Z., Liu S., Xiao Y., Sun M., Su J. (2014). Characterization of fly ash from a circulating fluidized bed incinerator of municipal solid waste. Environ. Sci. Pollut. Res..

[18] Xiao X., Yang H., Zhang H., Lu J., Yue G. (2005). Research on Carbon Content in Fly Ash from Circulating Fluidized Bed Boilers. Energy Fuels.

[19] Zhang W., Liu X., Zhang Z., Wang Y., Xue Y., Hao X., Lu Y. (2021). Circulating Fluidized Bed Fly Ash Mixed Functional Cementitious Materials: Shrinkage Compensation of f-CaO, Autoclaved Hydration Characteristics and Environmental Performance. Materials.

[20] Zhai W., Ding J., An X., Wang Z. (2020). An optimization model of sand and gravel mining quantity considering healthy ecosystem in Yangtze River, China. J. Clean. Prod..

[21] Holušová A., Poledniková Z., Vaverka L., Galia T. (2023). Spatiotemporal dynamics and present perception of gravel bars in natural and regulated environments. Sci. Total Environ..

[22] (2023). Technical Guide for Construction of Cement Stabilized Macadam Base of Expressway.

[23] (2015). Technical Guidelines for Construction of Highway Roadbases.

[24] He K.-W., Lu Z.-Y., Li J., Song K.-P. (2014). Comparative study on properties of circulating fluidized bed combustion ash and slag. Wuhan Ligong Daxue Xuebao J. Wuhan Univ. Technol..

[25] Chen D., Chen X., Liu Y., He Y. (2015). Expansion of circulating fluidized bed fly ash and control measures. Adv. Eng. Sci..

[26] Zhang W., Gu J., Zhou X., Li Y., Wang Y., Xue Y., Liu X. (2021). Circulating fluidized bed fly ash based multi-solid wastes road base materials: Hydration characteristics and utilization of SO_3_ and f -CaO. J. Clean. Prod..

[27] Li D., Sun R., Wang D., Ren C., Fang K. (2021). Study on the pozzolanic activity of ultrafine circulating fluidized-bed fly ash prepared by jet mill. Fuel.

[28] Han D.-X., Yan R.-Z., Li Q., Zhao S.-L., Jia K.-R. (2022). Expansion Performance of Hardened Cement Paste with Circulating Fluidized Bed Fly Ash under Different Curing Condition. Bull. Chin. Ceram. Soc..

[29] Zhou M.-K., Rao K., Meng X.-Y., Wang Y.-Q. (2024). Preparation and High-Strength Micro-Expansion Mechanism of CFB Fly Ash Compaction Slurry. Bull. Chin. Ceram. Soc..

[30] Wang J., Wang Y., Wang Y.-Q., Wang C.-P., Ye S.-M., Gao P. (2023). CBR Characteristics and Expansion Mechanism of Circulating Fluidized Bed Fly Ash. Bull. Chin. Ceram. Soc..

[31] Du X.-Y., Chen X., Zhou M.-K., Zhang H.-Y., Yang Y., Wang Y.-D. (2023). Effects of Sulfate-Containing Solid Wastes on Hydration of Portland Cement. Bull. Chin. Ceram. Soc..

[32] Wang X., Ni W., Li J., Zhang S., Hitch M., Pascual R. (2019). Carbonation of steel slag and gypsum for building materials and associated reaction mechanisms. Cem. Concr. Res..

[33] Soukal F., Ptacek P., Opravil T., Havlica J., Brandstetr J., Ovecka Z. (2014). The synthesis and characterization of an expansive additive for M-type cements. J. Therm. Anal. Calorim..

[34] (2017). Utilization of flue gas desulfurization gypsum for producing calcium sulfoaluminate cement. J. Clean. Prod..

[35] (2024). Standard Test Method for Relative Density (Specific Gravity) and Absorption of Coarse Aggregate.

[36] Yang Y., Zhang Q., Shu X., Wang X., Ran Q. (2022). Influence of Sulfates on Formation of Ettringite during Early C3A Hydration. Materials.

[37] Kharchenco I., Alekseev V. (2019). Effect of ettringite morphology on the properties of expanding cement systems. E3S Web Conf..

[38] Cody A.M., Lee H., Cody R.D., Spry P.G. (2004). The effects of chemical environment on the nucleation, growth, and stability of ettringite [Ca_3_Al(OH)_6_]2(SO_4_)3·26H_2_O. Cem. Concr. Res..

[39] Lubej S., Ivani A., Rudolf R., Anel I. (2012). Influence of Delayed Ettringite Formation on the Mechanical Properties of Aerated Concrete. Mater. Technol..

[40] Guo X., Ma H., Zhou J., Cheng W., Ba M. (2024). Stabilization mechanism of hexavalent chromium ions in Portland cement-based materials. Case Stud. Constr. Mater..

[41] Park H., Jeong Y., Jun Y., Jeong J.-H., Oh J.E. (2016). Strength enhancement and pore-size refinement in clinker-free CaO-activated GGBFS systems through substitution with gypsum. Cem. Concr. Compos..

[42] Sun Y., Bai F., Lü X., Jia C., Wang Q., Guo M., Li Q., Guo W. (2015). Kinetic study of Huadian oil shale combustion using a multi-stage parallel reaction model. Energy.

[43] Wei Z., Wang B., Falzone G., La Plante E.C., Okoronkwo M.U., She Z., Oey T., Balonis M., Neithalath N., Pilon L. (2018). Clinkering-free cementation by fly ash carbonation. J. CO2 Util..

[44] Jaschik J., Jaschik M., Warmuzinski K. (2016). The utilisation of fly ash in CO_2_ mineral carbonation. Chem. Process Eng..

[45] Pan S.-Y., Chang E.E., Chiang P.-C. (2012). CO_2_ Capture by Accelerated Carbonation of Alkaline Wastes: A Review on Its Principles and Applications. Aerosol Air Qual. Res..

[46] Thiery M., Villain G., Dangla P., Platret G. (2007). Investigation of the carbonation front shape on cementitious materials: Effects of the chemical kinetics. Cem. Concr. Res..

[47] Pérez-Maqueda L.A., Criado J.M., Sánchez-Jiménez P.E. (2006). Combined Kinetic Analysis of Solid-State Reactions:  A Powerful Tool for the Simultaneous Determination of Kinetic Parameters and the Kinetic Model without Previous Assumptions on the Reaction Mechanism. J. Phys. Chem. A.

[48] Vance K., Falzone G., Pignatelli I., Bauchy M., Balonis M., Sant G. (2015). Direct Carbonation of Ca(OH)_2_ Using Liquid and Supercritical CO_2_: Implications for Carbon-Neutral Cementation. Ind. Eng. Chem. Res..

[49] Xian X., Zhang D., Lin H., Shao Y. (2022). Ambient pressure carbonation curing of reinforced concrete for CO_2_ utilization and corrosion resistance. J. CO2 Util..

[50] Auroy M., Poyet S., Le Bescop P., Torrenti J.-M., Charpentier T., Moskura M., Bourbon X. (2018). Comparison between natural and accelerated carbonation (3% CO_2_): Impact on mineralogy, microstructure, water retention and cracking. Cem. Concr. Res..

[51] Li B., Sun Z., Hu K., Yang J. (2020). Influence of carbonation on the volume change of hardened cement pastes. Constr. Build. Mater..

[52] Matsushita F., Aono Y., Shibata S. (2004). Calcium silicate structure and carbonation shrinkage of a tobermorite-based material. Cem. Concr. Res..

[53] Li Y., Zeng H., Zhang H. (2023). Atomistic simulations of nucleation and growth of CaCO3 with the influence of inhibitors: A review. Mater. Genome Eng. Adv..

[54] Nishikawa T., Suzuki K., Ito S., Sato K., Takebe T. (1992). Decomposition of synthesized ettringite by carbonation. Cem. Concr. Res..

[55] Morandeau A., Thiéry M., Dangla P. (2014). Investigation of the carbonation mechanism of CH and C-S-H in terms of kinetics, microstructure changes and moisture properties. Cem. Concr. Res..

[56] Xu N., Ma S., Wang N., Feng Y., Li Y., Ren K., Bai S. (2024). Adding hydrated lime for regulating hydration and carbonation properties of circulating fluidized bed boiler fly ash. Mater. Today Commun..

[57] Wang X., Wei X., Ni W. (2024). Impacts of hydration degree of steel slag on its subsequent CO_2_ capture behaviors and mechanical performances of prepared building materials. Constr. Build. Mater..

[58] García Lodeiro I., Macphee D.E., Palomo A., Fernández-Jiménez A. (2009). Effect of alkalis on fresh C–S–H gels. FTIR analysis. Cem. Concr. Res..

[59] Borrachero M.V., Payá J., Bonilla M., Monzó J. (2008). The use of thermogravimetric analysis technique for the characterization of construction materials. J. Therm. Anal. Calorim..

[60] Chen S., Yuan H. (2022). Characterization and optimization of eco-friendly cementitious materials based on titanium gypsum, fly ash, and calcium carbide residue. Constr. Build. Mater..

[61] Du H., Xu D., Li X., Li J., Ni W., Li Y., Fu P. (2022). Application of molten iron desulfurization slag to replace steel slag as an alkaline component in solid waste-based cementitious materials. J. Clean. Prod..

[62] Qing L., Chuanming L., Huili S., Junxiang W., Xianjun L. (2023). Use of activated quartz powder as an alkaline source for producing one-part Ca-rich slag based cementitious materials: Activation mechanism, strength, and hydration reaction. J. Build. Eng..

[63] Feng P., Bullard J.W. (2016). Factors Influencing the Stability of AFm and AFt in the Ca-Al-S-O-H System at 25 °C. J. Amer. Ceram. Soc..

[64] Matschei T., Lothenbach B., Glasser F.P. (2007). The AFm phase in Portland cement. Cem. Concr. Res..

[65] Lin R.-S., Oh S., Du W., Wang X.-Y. (2022). Strengthening the performance of limestone-calcined clay cement (LC3) using nano silica. Constr. Build. Mater..

[66] Zhang G., Wu C., Hou D., Yang J., Sun D., Zhang X. (2021). Effect of environmental pH values on phase composition and microstructure of Portland cement paste under sulfate attack. Compos. Part B Eng..

[67] Helmi M., Hall M.R., Stevens L.A., Rigby S.P. (2016). Effects of high-pressure/temperature curing on reactive powder concrete microstructure formation. Constr. Build. Mater..

[68] Hu Z., Wyrzykowski M., Griffa M., Scrivener K., Lura P. (2020). Young’s modulus and creep of calcium-silicate-hydrate compacts measured by microindentation. Cem. Concr. Res..

[69] Zhao J., Sun C., Wang Q., Shen X., Lu L. (2022). Thermodynamic, mechanical, and electronic properties of ettringite and AFm phases from first-principles calculations. Constr. Build. Mater..

[70] Huang D., Liu H., Hou M.-Q., Xie M.-Y., Lu Y.-F., Liu L., Yi L., Cui Y.-J., Li Y., Deng L.-W. (2017). Elastic properties of CaCO_3_ high pressure phases from first principles. Chin. Phys. B.

[71] Laveglia A., Sambataro L., Ukrainczyk N., De Belie N., Koenders E. (2022). Hydrated lime life-cycle assessment: Current and future scenarios in four EU countries. J. Clean. Prod..

[72] Xu N., He Y., Sa R., Wang N., Yang Y., Ma S. (2025). Optimizing Mechanical Properties and Environmental Benefits of CFBFA Composite Gravels Through Gypsum, Hydrated Lime Addition, and CO_2_ Carbonation Curing. Solids.

[73] Doleželová M., Scheinherrová L., Vimmrová A. (2021). Study of gypsum composites with fine solid aggregates at elevated temperatures. Sci. Eng. Compos. Mater..

[74] Zhu P., Li H., Liu H., Yan X., Wang X., Chen C. (2022). Effect of CO_2_ Curing on the Physical Properties of Recycled Coarse Aggregate with Different Attached Mortar Contents. J. Wuhan Univ. Technol. Mater. Sci. Ed..

[75] Li C., Cui S., Nie Z., Gong X., Wang Z., Itsubo N. (2015). The LCA of portland cement production in China. Int. J. Life Cycle Assess..

[76] Batuecas E., Ramón-Álvarez I., Sánchez-Delgado S., Torres-Carrasco M. (2021). Carbon footprint and water use of alkali-activated and hybrid cement mortars. J. Clean. Prod..

